# Probing the Capsid: pH-Driven Gating at the AAV 5-Fold Pore and Its Role in Peptide Ligand Binding

**DOI:** 10.3390/pharmaceutics18091053

**Published:** 2026-08-25

**Authors:** Arianna Minzoni, Benjamin Bobay, Shriarjun Shastry, Eduardo Barbieri, Brandon Brino, Crystal Collazo, Shizuo Kamita, Danni Wang, Ciera Khuu, Alexander Polgar, Joseph Siino, Sushmita Koley, Peyton Russelburg, Mark Snyder, Christopher Belisle, Michael Daniele, Stefano Menegatti

**Affiliations:** 1 Department of Chemical and Biomolecular Engineering, NC State University, 911 Partners Way, Raleigh, NC 27606, USA; 2 North Carolina Viral Vector Initiative in Research and Learning (NC-VVIRAL), NC State University, 911 Oval Dr, Raleigh, NC 27695, USA; 3 Duke University NMR Center, Department of Chemistry, Duke University Medical Center, 308 Research Dr, Durham, NC 27710, USA; 4 Biomanufacturing Training and Education Center (BTEC), NC State University, 850 Oval Dr, Raleigh, NC 27695, USA; 5 Health Research Institute, Michigan Technological University, 1400 Townsend Drive, Houghton, MI 49931, USA; 6 Bio-Rad Laboratories, 1000 Alfred Nobel Drive, Hercules, CA 94547, USA; 7 Department of Electrical and Computer Engineering, NC State University, Raleigh, NC 27695, USA; 8 Lampe Joint Department of Biomedical Engineering, NC State University, Raleigh, NC 27695, USA; 9 Chromagenix LLC, 1791 Varsity Drive, Raleigh, NC 27606, USA

**Keywords:** adeno-associated virus, capsid proteins, mixed-mode chromatography, molecular dynamics, downstream processing

## Abstract

**Background/Objectives:** Adeno-associated virus (AAV) capsids undergo pH-dependent conformational gating at the 5-fold symmetry pore, but how these structural dynamics shape serotype-specific behavior and affinity-ligand recognition remains unclear, particularly for the clinically important serotypes AAV8 and AAV9. This study aimed to establish a pH-resolved structural framework linking 5-fold pore dynamics to peptide-ligand recognition and to translate this framework into sequence-based design principles for affinity capture of gene therapy vectors. **Methods:** AAV8 and AAV9 5-fold capsid assemblies were subjected to 500 ns molecular dynamics simulations under acidic (pH 5), neutral (pH 7), and basic (pH 9) conditions, with analysis of pore volume, inter-residue contact networks, electrostatic potential, and solvent-accessible surface area. In parallel, affinity chromatography using three mixed-mode peptide ligands (RVVAVYRI, TTFRAHHI, and TYHHHHII) was performed on clarified HEK293 lysates containing AAV8 or AAV9, with capsid yield, host-cell-protein clearance, and transduction activity assessed by ELISA, SEC-HPLC, and flow-cytometry-based transduction assays. **Results:** AAV8 displayed a heterogeneous, bimodal pore conformational landscape at pH 7, whereas AAV9 exhibited a discrete gate-like transition with maximal pore constriction at physiological pH; both serotypes showed pore-proximal contact remodeling with distinct network topologies. Experimentally, TYHHHHII achieved the highest selectivity for genome-containing capsids at pH 7, with transduction activity enrichment factors of 2.82 (AAV8) and 5.61 (AAV9), while TTFRAHHI provided the broadest operational pH range for bulk capsid recovery. **Conclusions:** These findings establish a structural framework linking pH-dependent pore dynamics to affinity ligand recognition and suggest practical sequence-design rules for ligand engineering: clustered histidines for neutral-pH selectivity, Arg-containing motifs for broad-pH robustness, and aromatic or hydrophobic residues for reinforcement of capsid binding.

## 1. Introduction

With over 20 regulatory approvals and a rapidly expanding pipeline of investigational treatments, adeno-associated virus (AAV) vectors have become the dominant platform for in vivo gene therapy. A persistent challenge in AAV manufacturing, however, is the co-production of empty capsids devoid of therapeutic payload, which must be removed to meet regulatory potency thresholds and reduce immunogenic burden. Among the naturally occurring serotypes, AAV8 and AAV9 are of particular clinical relevance: AAV8 exhibits robust hepatocyte tropism widely exploited for liver-directed gene delivery, while AAV9 demonstrates broad systemic transduction and an exceptional capacity to cross the blood–brain barrier. Despite their shared icosahedral architecture and high capsid protein sequence identity (83%), AAV8 and AAV9 differ substantially in receptor utilization, intracellular trafficking, immunological profiles, and tissue selectivity, which are encoded in the fine structural details of their capsid surfaces [1,2,3], [4] p. 9, [5].

The 60-subunit T = 1 icosahedral capsid of AAV is assembled from three overlapping viral proteins (VP1, VP2, and VP3) that adopt an eight-stranded β-barrel fold connected by variable surface loops (VRI through VRIX). These loops constitute the major determinants of receptor binding, antigenic identity, and tissue tropism. At the 5-fold symmetry axes, the capsid subunits assemble to form a narrow cylindrical channel—the 5-fold pore—conserved across AAV serotypes and formed primarily by the DE-loop ribbon and adjacent β-strand D. First characterized in AAV2 by mutagenesis, these pores measure approximately 1.2 nm in diameter and adopt a camera-shutter-like conformation defined by pore-lining residues 217–223 and 322–338. Functional studies established that the 5-fold pore serves as a conduit for genome packaging by the Rep helicase and, critically, for the externalization of the VP1 N-terminal phospholipase A2 (PLA2) domain required for endosomal escape [6,7,8].

While the structural basis of the 5-fold pore is well characterized in AAV2 [7,9], comparatively less is known about how pore architecture and dynamic behavior diverge between AAV8 and AAV9—two serotypes with distinct endosomal trafficking kinetics and intracellular uncoating efficiencies. Crystal structures of AAV8 revealed that its 5-fold pore retains the canonical ribbon channel geometry, but with a notable asparagine-to-serine substitution at position 223 that subtly alters the electrostatic environment of the pore lumen [6,10,11]. In contrast, the AAV9 5-fold axis is flanked by distinctive DE-loop and VRII configurations that alter the topology of the pore entrance. Recent cryo-EM analyses of AAV9 virus-like particles (VLPs) have demonstrated that the 5-fold pores adopt a predominantly closed conformation under acidic pH conditions—consistent with endosomal pH encountered during trafficking—whereas related parvoviral capsids show pore opening under equivalent conditions. These pH-dependent rearrangements in AAV9 extend beyond the 5-fold axis to encompass reordering of the DE and HI loops, suggesting a dynamic gating mechanism that may regulate both the timing and efficiency of VP1 externalization [8,12,13].

The comparative structural analysis across the AAV serotype family has further highlighted serotype-specific differences in variable regions immediately flanking the 5-fold pore—particularly VRII in AAV9—which create divergent electrostatic and steric environments at the channel entrance. These differences may influence genome encapsidation geometry and pore accessibility during uncoating. To date, cryo-EM studies of individual serotypes and mutagenesis analyses of the AAV2 pore have provided partial structural frameworks; however, none have delivered a direct, pH-resolved, side-by-side comparison of the 5-fold pore in AAV8 and AAV9 at sufficient resolution to delineate pore-lining residue identities, hydrogen-bonding networks, and pH-responsive conformational states [14,15,16]. Such a comparison is timely given growing interest in engineering the 5-fold channel to modulate immunogenicity, improve genome retention, and enhance intracellular uncoating efficiency. Critically, because peptide ligands engage the capsid surface through the same electrostatic and hydrophobic contacts that govern pore dynamics, a pH-resolved structural model of the 5-fold interface provides a rational design framework for affinity ligand design—directly linking structural biology to downstream bioprocessing.

Here, we report pH-resolved MD simulations of the 5-fold pore in AAV8 and AAV9 pentameric assemblies under acidic, neutral, and basic conditions, identifying conserved and divergent features that underlie serotype-specific differences in pore gating behavior and presenting a structural model for pH-dependent regulation of the 5-fold channel. Building on this structural framework, we evaluate experimentally how pH and ionic strength modulate the binding of three histidine-rich mixed-mode peptide ligands [17]—RVVAVYRI, TTFRAHHI, and TYHHHHII—to AAV8 and AAV9 capsids across pH 5–9, demonstrating that histidine-rich peptides function as programmable molecular switches capable of either maximizing capsid recovery or selectively enriching genome-containing particles within a single chromatographic step, and establishing sequence-based design principles for their rational development as affinity capture reagents for gene therapy manufacturing.

## 2. Materials and Methods

### 2.1. Materials

Fluorenylmethoxycarbonyl (Fmoc)-protected amino acids Fmoc-Gly-OH, Fmoc-Ser(tBu)-OH, Fmoc-Ile-OH, Fmoc-Ala-OH, Fmoc-Phe-OH, Fmoc-Tyr(tBu)-OH, Fmoc-Asp(OtBu)-OH, Fmoc-His(Trt)-OH, Fmoc-Arg(Pbf)-OH, Fmoc-Lys(Boc)-OH, Fmoc-Glu(OtBu)-OH, Fmoc-Thr(tBu)-OH, Fmoc-Val-OH, and Fmoc-Leu-OH, the coupling agent Azabenzotriazole Tetramethyl Uronium Hexafluorophosphate (HATU), diisopropylethylamine (DIPEA), piperidine, and trifluoroacetic acid (TFA) were procured from ChemImpex International (Wood Dale, IL, USA). Triisopropylsilane (TIPS), 1,2-ethanedithiol (EDT), anisole, Bis-Tris Propane, and sodium acetate were sourced from MilliporeSigma (Burlington, MA, USA). N,N′-dimethylformamide (DMF), dichloromethane (DCM), methanol, N-methyl-2-pyrrolidone (NMP), phosphate-buffered saline (PBS) tablets, hydrochloric acid, Bis-Tris, Tris-base, magnesium chloride, sodium chloride, Pluronic F-68, sodium azide, potassium chloride, Gibco Viral Production Cells Prototype 2.0, AAV-MAX Enhancer, Viral-Plex Complexation Buffer, Transfection Reagent, AAV-MAX Lysis Buffer, Dulbecco’s Modified Eagle Medium (DMEM), and fetal bovine serum (FBS) were obtained from ThermoFisher Scientific (Waltham, MA, USA). Benzonase endonuclease (250 KU) was obtained from Syd Lab (Woodbridge, NJ, USA). HT1080 cells were obtained from ATCC (Manassas, VA, USA). Tricorn 5/50 chromatography columns (5 mm I.D., 50 mm length) were obtained from Cytiva (Uppsala, Sweden). AAV8 and AAV9 capsid ELISA kits were obtained from PROGEN (Wayne, PA, USA). HEK293 host cell protein (HCP) ELISA kits (Generation 3) were acquired from Cygnus Technologies (Southport, NC, USA). The BioResolve SEC mAb column (200 Å, 2.5 μm, 7.8 × 300 mm) was obtained from Waters Corporation (Milford, MA, USA). For AAV production, the plasmids pRC2-AAV8 and pRC2-AAV9 were sourced from Addgene (Watertown, MA, USA), and pALD-Helper and pALD-ITR-GFP were obtained from Aldevron (Madison, WI, USA).

### 2.2. Production and Harvest of AAV from HEK293F Cell Cultures

Gibco Viral Production Cells 2.0 were seeded at 3.0 × 10^6^ cells/mL and supplemented with AAV-MAX Enhancer at 1% *v*/*v*, then incubated in a humidified shaker incubator (37 °C, 8% CO_2_, 120 rpm). The transfection mixture was prepared by combining the pRC2 plasmid (encoding Rep and Cap for the desired AAV serotype), the pHelper plasmid (adenoviral helper functions), and the pAAV-GFP plasmid (packaged GFP transgene) at the molar DNA ratio of 2:0.5:1 (pRC2:pHelper:pAAV-GFP), for a total DNA concentration of 2.0 μg/mL of culture. This plasmid cocktail was diluted in ViralPlex Complexation Buffer to 10% of the culture volume. In parallel, Transfection Booster and Transfection Reagent were mixed at 0.3% and 0.6% of the culture volume, respectively. The DNA/ViralPlex and Booster/Reagent solutions were combined, incubated at room temperature for 20 min to allow complex formation, and then added to the Viral Production Cells. After 72 h, AAV-MAX Lysis Buffer was added at 10% of the culture volume to lyse cells. The resulting lysate was supplemented with MgCl_2_ to 2 mM and benzonase endonuclease to 50 U/mL, then incubated at 37 °C for 2 h to degrade residual host-cell nucleic acids. The crude lysate was then clarified by centrifugation at 4500× *g* for 30 min, and the supernatant containing AAV particles was collected for downstream processing.

### 2.3. AAV Purification from Clarified HEK293F Cell Lysates Using Peptide-Functionalized Resins

The candidate peptide ligands were synthesized and conjugated to the resin at the density of ~0.05 mmol of peptide per mL of resin as described in prior work [18,19,20,21]. Aliquots of 0.5 mL of peptide-functionalized resin were flow-packed into Tricorn 5/50 chromatography columns (5 mm I.D., 50 mm length). The columns were initially equilibrated with 20 column volumes (CVs) of binding buffer (10 mM Bis-Tris buffer, 20 mM NaCl, 0.1% Pluronic-F68 at pH 7.0) at the residence time (RT) of 1 min. The clarified lysate (AAV8 capsid titer: 2.2 × 10^12^–3.4 × 10^12^ vp/mL; HCP titer: 0.28 mg/mL, AAV9 capsid titer: 1.5 × 10^12^–3.0 × 10^12^ vp/mL; HCP titer: 0.32 mg/mL) was loaded on the column at the RT of 3 min. After loading, the column was washed with 20 CVs of binding buffer. The AAVs were eluted in three steps: 10 CVs of 10 mM Bis-Tris buffer, 0.2 M MgCl_2_, 0.1% Pluronic-F68, at pH 6.0 at the RT of 2 min, followed by 10 CVs of 10 mM Bis-Tris buffer, 0.4 M MgCl_2_, 0.1% Pluronic-F68, at pH 6.0 at the RT of 2 min, and 10 mM Bis-Tris buffer, 1.0 M MgCl_2_, 0.1% Pluronic-F68, at pH 6.0 at the RT of 2 min. The flow-through, wash, strip, and elution fractions were collected for subsequent analysis, as described in Section 2.4, Section 2.5, Section 2.6 and Section 2.7.

### 2.4. Quantification of AAV Capsids via Serotype-Specific AAV ELISA Kits

The AAV capsid titers in load, flow-through, wash, and elution fractions were measured using serotype-specific ELISA kits (PROGEN, Wayne, PA, USA) according to the manufacturer’s instructions. Briefly, appropriately diluted samples were incubated on pre-coated plates, followed by detection with serotype-specific antibodies and colorimetric readout, and titers were calculated from standard curves generated with reference AAV standards.

### 2.5. Quantification of HEK293 HCPs

The HEK293 HCP concentration in load and elution fractions were measured using G3 HEK293 HCP ELISA kits (Cygnus Technologies, Leland, NC, USA) according to the manufacturer’s instructions.

### 2.6. Size Exclusion Chromatography (SEC) on HPLC

The collected fractions were analyzed by analytical SEC using a BioResolve SEC mAb column (200 Å, 2.5 μm, 7.8 × 300 mm; Waters, Milford, MA, USA). The column was operated with a 40 min isocratic method using a mobile phase of 50 mM sodium phosphate, 200 mM potassium chloride, 0.05% (*w*/*v*) sodium azide, and 0.01% (*v*/*v*) Pluronic F68 at pH 7.0. A 10 μL sample volume was injected for each run, and the effluent was monitored by UV absorbance at 260 nm and 280 nm, as well as by fluorescence detection with excitation at 280 nm and emission at 350 nm. *Note:* SEC-HPLC quantifies all capsid-sized material eluting within the capsid retention window by intrinsic protein fluorescence; this technique is not serotype-specific and does not discriminate assembled capsids from co-eluting species of similar hydrodynamic size, and may slightly over-report if aggregated HCPs or hcDNA co-elutes with the capsid peak. The capsid ELISA employs a conformation-dependent antibody that binds only assembled particles presenting an intact quaternary epitope, and therefore under-reports where that epitope is perturbed (e.g., following exposure to the MgCl_2_ elution buffer or to acidic loading pH), even when the particle remains intact. Across the conditions with measurable binding the two methods agree, with neither method systematically higher. Accordingly, in this study the capsid ELISA is used as the primary yield metric, being capsid- and serotype-specific, and SEC-HPLC for the fraction-resolved mass balance across load, flow-through, wash, elution and strip, being applicable rapidly and uniformly to every fraction.

### 2.7. Transduction Assay Through Fluorescence Flow Cytometry (FFC)

HT1080 cells were maintained in DMEM supplemented with 10% *v*/*v* FBS at 37 °C and 5% CO_2_ and passaged until reaching 80–90% confluence. Cells were then seeded in 96-well plates at 5000 cells per well in complete DMEM and cultured for 24 h to reach a density of 10,000 cells/mL. AAV samples from clarified HEK293 lysates and column eluates were serially diluted in serum- and antibiotic-free DMEM, and 100 μL of each dilution was added to the HT1080 monolayers. After 24 h, the inoculum was replaced with fresh DMEM containing 10% *v*/*v* FBS, and cells were incubated for an additional 24 h. The fraction of GFP-positive (GFP^+^) cells was quantified on a CytoFLEX flow cytometer (Beckman Coulter, Brea, CA, USA), and transduction units per milliliter (TU/mL) were calculated from the percentage of GFP^+^ cells and the number of target cells according to Equation (1).

(1) $$\mathrm{Activity}\;\left(\frac{\mathrm{TU}}{\mathrm{mL}}\right)=\frac{N_{\mathrm{HT}1080}\;\times \;{\%\mathrm{GFP}}^{+}}{V\;\times \;\mathrm{DF}}$$

wherein N_HT1080_ is the number of cells incubated with the diluted AAV sample, %GFP^+^ is the percentage of cells expressing GFP, V is the volume of the diluted AAV sample, and DF is the dilution factor. The Activity Enrichment Factor, defined as the ratio of the transduction efficiency (TE) of the eluted AAV samples to that of the input (feed) AAV samples, was calculated using Equation (2).

(2) $$\mathrm{Activity}\;\mathrm{Enrichment}\;\mathrm{Factor}=\frac{\frac{[\mathrm{Activity}\;(\frac{\mathrm{TU}}{\mathrm{mL}})]}{[\mathrm{AAV}\;(\frac{\mathrm{cp}}{\mathrm{mL}})]}\;(\mathrm{Elution})}{\frac{[\mathrm{Activity}\;(\frac{\mathrm{TU}}{\mathrm{mL}})]}{[\mathrm{AAV}(\frac{\mathrm{cp}}{\mathrm{mL}})]}\;(\mathrm{Feed})}$$

### 2.8. MD Simulation of 5-Mer AAVs

Molecular dynamics (MD) simulations of 5-mer AAV capsid assemblies were performed for AAV8 (PDB ID: 6V10) and AAV9 (PDB ID: 7WJW). Protonation states corresponding to each pH condition were assigned prior to simulation using standard pKa prediction protocols using the PDB2PQR server with AMBER parameterization [22], and systems were simulated independently under these conditions. All simulations were carried out using GROMACS version 2020.5 with the AMBER03 force field and the flexible SPC water model [23,24]. For each serotype, a single VP1 5-mer was placed in a dodecahedral box of explicit solvent with periodic boundary conditions, maintaining a minimum distance of 1.0 nm between the protein and the box edge. Systems were neutralized with counterions, and long-range electrostatics were treated using the particle mesh Ewald method with a real-space cutoff of 1.0 nm; Lennard–Jones interactions were treated with the same cutoff.

Energy minimization was performed using the steepest-descent algorithm until the maximum force fell below 1000 kJ/mol/nm. Following minimization, systems were equilibrated in two phases: (i) constant volume and temperature (NVT) equilibration at 300 K using a V-rescale thermostat, followed by (ii) constant pressure and temperature (NPT) equilibration at 300 K and 1 bar using a Berendsen barostat. All covalent bonds were constrained using the LINCS algorithm, with a time step of 4 fs throughout. Production MD simulations were carried out for 500 ns under NPT conditions using a Parrinello–Rahman barostat and V-rescale thermostat. Position restraints were removed before production. Nonbonded interaction cutoffs were maintained at 1.0 nm unless otherwise noted. Trajectory stability was assessed using Cα root mean square deviation (RMSD), radius of gyration (Rg), and center-of-mass (COM) analysis. All metrics stabilized within the first 100 ns, and subsequent analyses were performed on the final 400 ns of each trajectory (Figure S10). All structural images were made with PyMOl [25]. Because the molecular dynamics analyses are based on time-resolved trajectory sampling rather than independent biological replicates, robustness was assessed through equilibration and convergence metrics, including Cα-RMSD, radius of gyration, center-of-mass stability, and consistency across independent structural descriptors, rather than through replicate-based *p*-value testing.

### 2.9. Pore Volume Calculations

Pore volumes were measured using a custom voxel-based Python 3.9.21 script applied to each symmetric 5-mer PDB assembly extracted from the final 400 ns of the MD simulations. A cubic grid with 1.0 Å spacing (1 Å × 1 Å × 1 Å voxels) was used to interrogate the volume surrounding the capsid. The oligomer axis was determined from chain-centroid geometry and aligned consistently with pore-lining residue groups. The pore search region was constrained along the axial direction using residue-guided boundaries defined by residues 220–224 and 333–339 (Cα atoms), with an additional ±1.5 Å padding to ensure complete coverage of the pore region. Radially, the pore was confined to the central lumen using the 25th percentile of radial distances of pore-lining residues, without additional radial expansion. Protein-occupied space was defined using van der Waals radii augmented by a 1.4 Å solvent probe radius. Voxels not occupied by protein and belonging to the connected empty-space component centered on the pore axis were classified as pore voxels. The pore volume was calculated as the total number of pore voxels multiplied by the voxel volume.

### 2.10. Definition of Inter-Residue Contacts

Inter-residue contacts were computed for each frame using a distance-based criterion implemented in custom Python scripts. Heavy atom pairs between residues were evaluated using distance data derived from PyMOL 3.0.4 across all structures from the final 400 ns of the simulations [25]. A contact was defined when any interatomic distance between two residues was ≤4.0 Å. Contacts were restricted to inter-chain interfaces to capture interactions between capsid subunits. Specifically, contacts involving chain A and its neighboring chains (B and E) were analyzed to characterize interactions across the 5-fold assembly. For each frame, atom-level contacts were reduced to residue–residue pairs, with multiple atom contacts collapsed into a single occurrence per frame. Contact frequency for each residue pair was accumulated across all frames and normalized by the total number of frames (Equation (3)) to yield contact occupancy:

(3) $$\mathrm{Occupancy}=\frac{\mathrm{number}\;\mathrm{of}\;\mathrm{frames}\;\mathrm{with}\;\mathrm{contact}}{\mathrm{total}\;\mathrm{number}\;\mathrm{of}\;\mathrm{frames}}$$

To reduce noise and facilitate visualization, residues were grouped into contiguous 5-residue bins, and this binning scheme was applied consistently across contact, SASA, and electrostatic analyses. Contacts were mapped from residue–residue pairs to bin–bin pairs, and binning was applied consistently across all datasets. Changes in contact occupancy between conditions were computed as:

$$\Delta occupancy=occupancy_{pH9}-occupancy_{pH5}$$

Positive values indicate contacts enriched at pH 9, whereas negative values indicate contacts enriched at pH 5. To investigate coordinated interfacial changes, a “shared-A” contact network was defined (the term ‘network’ is used conceptually to describe coordinated patterns of inter-residue connectivity), comprising chain A residue bins that simultaneously interact with adjacent chains B and E. These interactions were visualized using centered Sankey diagrams to represent three-chain connectivity. Residue bins identified in this analysis were mapped onto the capsid structure for structural interpretation.

### 2.11. Solvent Accessible Surface Area (SASA) Calculations

Residues were classified into three structural regions: the pore, the inside surface, and the outside surface. Initial classification was performed using a custom Python workflow that integrates geometric descriptors (radial distance from the capsid center, pore-axis distance, and residue orientation) with solvent accessibility metrics for full capsid structures of AAV8 and AAV9. These features were combined with SASA values to generate an initial classification, which was subsequently refined using a seeded, rule-based approach to ensure consistent identification of pore, inside, and outside residues across all capsid assembly chains (A–E). Custom Python scripts were used to assign residues to structural classes, propagate seed definitions across symmetric chains, and generate GROMACS-compatible index files for downstream analysis.

Solvent accessible surface area was calculated using the gmx sasa module in GROMACS [23]. Residue-level SASA values were extracted and processed using custom Python scripts that aligned residues across pH conditions and computed differences in solvent accessibility (ΔSASA) between conditions (pH 5–pH 7 and pH 9–pH 7). Residues were matched by residue number across all conditions to ensure consistent comparisons. Group-level statistics, including total SASA, mean per-residue SASA, and standard deviation, were computed for each structural region. To identify residues contributing most strongly to pH-dependent changes, the top 10% of residues ranked by absolute ΔSASA were selected using a percentile-based filtering approach. Interpretation of solvent exposure was based on relative changes in SASA across conditions rather than fixed absolute thresholds, allowing identification of residues that become more exposed or buried in a comparative manner.

### 2.12. Protein Electrostatics Calculations

Electrostatic potentials were analyzed using custom Python scripts applied to a randomly selected subset of 50 structures from the final 400 ns of each simulation. Preliminary analyses confirmed that this sampling size was sufficient to reproduce ensemble-averaged electrostatic trends. Electrostatic potentials were calculated using APBS [26], and resulting DX grids were mapped onto molecular coordinates. Residues were grouped into contiguous bins (5-residue bins spanning residues 200–750), and the mean electrostatic potential for each bin was calculated by sampling grid values at atomic positions within each bin. To identify regions exhibiting the largest electrostatic changes, the top 10% of residue bins ranked by absolute change in electrostatic potential relative to pH 7 were selected for further analysis. The resulting potentials were averaged over atoms and frames to yield ensemble-averaged values.

Electrostatic potentials were normalized within each system to enable comparison across serotypes and pH conditions. Heatmaps were generated using Matplotlib 3.9.4 with a symmetric red–white–blue diverging colormap centered at zero (red: negative potential, blue: positive potential, white: neutral). Color scaling was normalized per panel, using the maximum absolute potential value within each dataset; consequently, the pH rows within a given panel share a common scale and are directly comparable, whereas the scales of different panels are independent and magnitudes should not be compared between them. Residue bins were annotated by structural region (pore, interior, exterior) using predefined residue ranges. To identify representative structures, mean electrostatic potentials across the entire grid were computed for each frame, and structures with the highest and lowest values were selected as extremes of the electrostatic ensemble. APBS calculations were performed using a multigrid finite-difference approach with the following parameters: grid dimensions of 161 × 161 × 161, coarse grid length of 220 Å, fine grid length of 140 Å, dielectric constants of 2.0 (protein) and 78.5 (solvent), temperature of 298.15 K, solvent-accessible surface definition, spline-based charge discretization, and a solvent probe radius of 1.4 Å. The linearized Poisson–Boltzmann equation was solved at zero ionic strength and the electrostatic potentials were reported in units of kT/e. Zero ionic strength was chosen deliberately, in order to isolate the redistribution of charge produced by the change in protonation state from the Debye screening that would otherwise attenuate the computed potentials by a distance-dependent factor. The potentials reported here are therefore upper bounds on the magnitudes obtaining at the experimental ionic strength of 22.2–30.0 mM, which corresponds to a Debye length of approximately 1.8–2.1 nm; because every quantity derived from these calculations is either normalized within a dataset or expressed as a difference between pH conditions, screening would rescale the reported values without reordering the pH states or displacing the responsive residue bins. These analyses are correlational and are intended to identify relationships among electrostatic potential, inter-residue contacts, and pore dynamics rather than to establish direct causality. All electrostatic potentials were computed with APBS and all trajectories with GROMACS; the custom Python scripts used here perform geometric and statistical post-processing of these outputs—namely, voxel counting, residue binning, occupancy accumulation, and grid sampling at atomic positions—and introduce no additional physical approximation beyond the grid spacings and cut-offs stated above.

### 2.13. 5-Mer Trimer-Context AAV Model Construction and Peptide Binding Analysis

Trimer-context models were constructed from molecular dynamics (MD) snapshots of each AAV 5-mer simulation. For each snapshot, the simulated 5-mers (chains A–E) were duplicated and aligned in PyMOL [25] against two pre-generated symmetry-related neighboring 5-mer reference assemblies representing adjacent capsid environments. Structural alignment was performed using PyMOL cealign. For each MD snapshot, two copies were generated and independently aligned to the two neighboring symmetry-mate reference assemblies, yielding three aligned copies per snapshot corresponding to the central 5-mer and two symmetry-related neighboring 5-mers. The three aligned 5-mers were then merged into a single 15-chain trimer-context model representing the local capsid environment surrounding the peptide-binding region. The resulting trimer-context models served as the basis for all subsequent solvent-exposure, contact, and electrostatic calculations, each performed as above with the following exceptions. SASA calculations were performed using the Shrake–Rupley rolling-probe algorithm as implemented in FreeSASA, using a 1.4 Å probe radius to approximate a water molecule [27]. Per-residue SASA values were calculated for capsid:peptide interface residues in each snapshot, and averaged across analyzed frames [28]. To quantify burial associated with peptide binding or pH-dependent rearrangements, differential SASA (ΔSASA) values were calculated as the difference in residue accessibility between pH states. For residue-level summaries and regional analyses, residues were additionally grouped into 5-residue bins to facilitate comparison with contact-based analyses.

### 2.14. Statistical Analysis and Reporting

**Figure 1:**
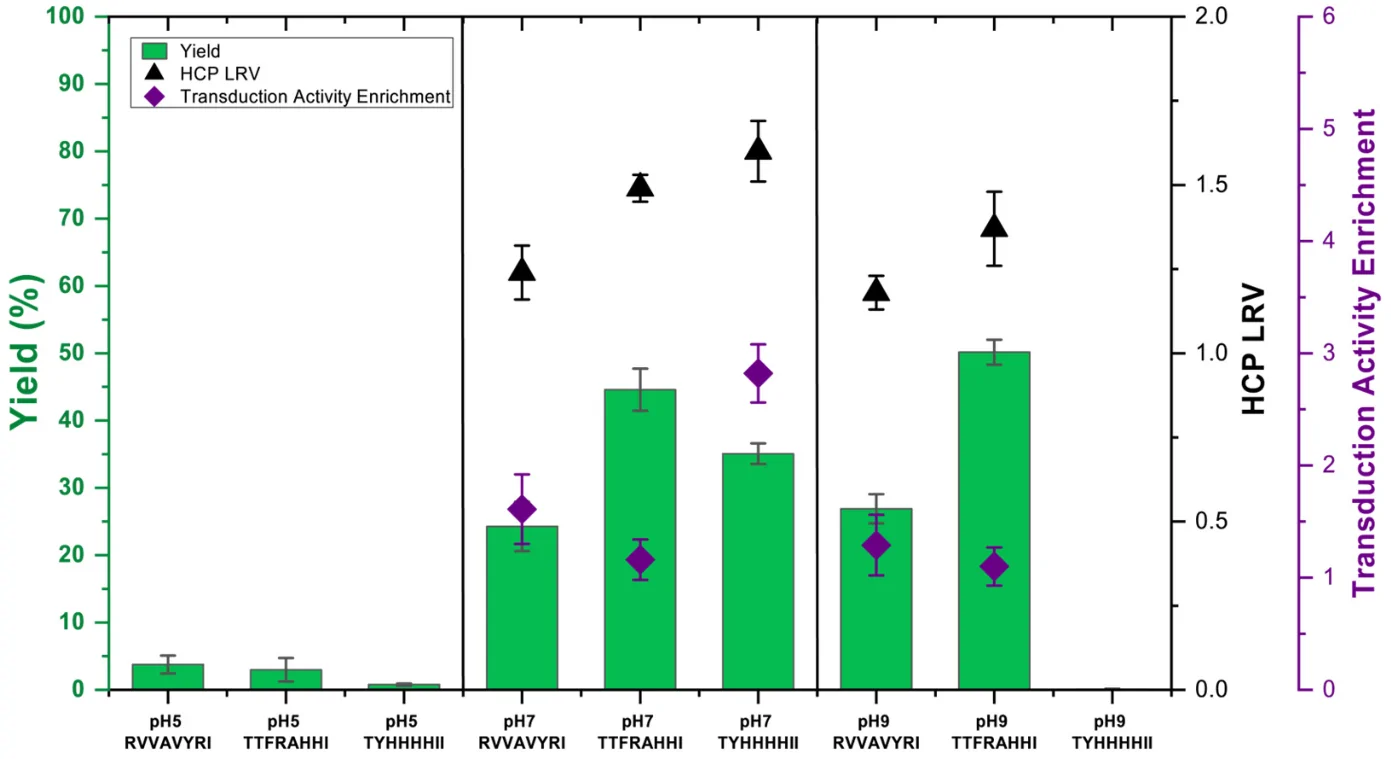
Capsid yield, enrichment of cell-transducing virions, and HCP reduction obtained by purifying AAV8 from clarified HEK293 cell lysates using peptide-functionalized resins. Each chromatographic condition was performed as a single run; the capsid ELISA, HCP ELISA, and transduction assay quantifications were performed in technical replicate (*n* = 3), and the values shown are the means of those replicates. The corresponding numerical values are reported in Table S3.

**Figure 2:**
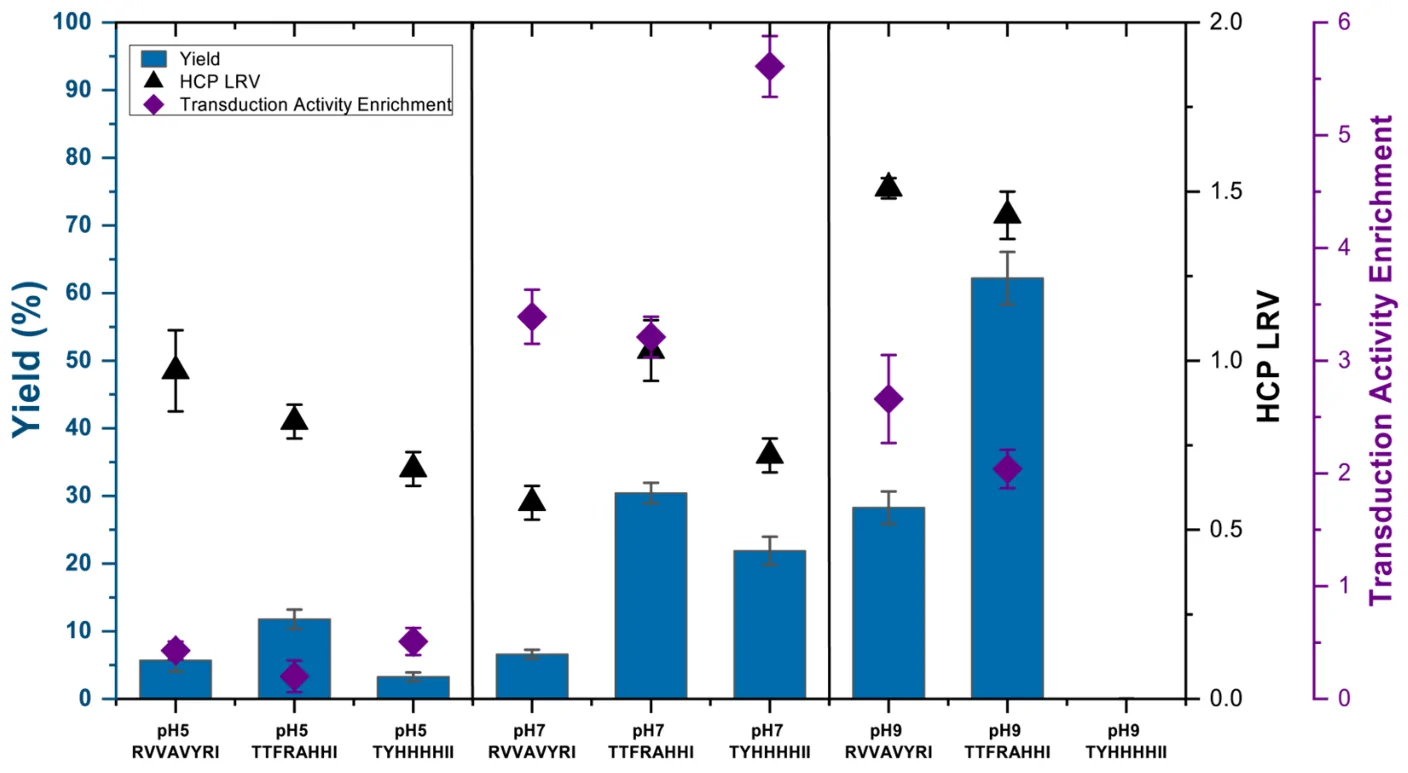
Capsid yield, enrichment of cell-transducing virions, and HCP reduction obtained by purifying AAV9 from clarified HEK293 cell lysates using peptide-functionalized resins. Each chromatographic condition was performed as a single run; the capsid ELISA, HCP ELISA, and transduction assay quantifications were performed in technical replicate (*n* = 3), and the values shown are the means of those replicates. The corresponding numerical values are reported in Table S4.

The chromatographic dataset reported in this study is a comparative screen spanning a full factorial matrix of eighteen conditions (two serotypes × three loading pH values × three peptide ligands). Each chromatographic condition was performed as a single run, and the analytical quantifications carried out on the resulting load, flow-through, wash, and elution fractions—capsid ELISA, HCP ELISA, SEC-HPLC, and flow-cytometry transduction assay—were performed in technical replicate (*n* = 3), with the values reported in Figure 1 and Figure 2 and in Tables S3 and S4 representing the mean of those replicates. Because each condition corresponds to a single chromatographic run, no inferential statistics or *p*-values are reported for the chromatographic data; the evidentiary weight of the experimental dataset rests on the consistency of the trends observed across the eighteen conditions rather than on within-condition replication. Statistical treatment of the molecular dynamics data follows a different logic and is described in Section 2.8: because trajectories sample a configurational ensemble rather than independent biological realizations, robustness was assessed through equilibration and convergence metrics and through the agreement of independent structural descriptors, not through replicate-based hypothesis testing.

### 2.15. Data and Code Availability

All physical quantities reported in this study were computed with established software: molecular dynamics with GROMACS 2020.5, protonation state assignment with PDB2PQR 3.7.1, solvent-accessible surface areas with the GROMACS gmx sasa module and with FreeSASA 2.1.2, electrostatic potentials with APBS, and structural superposition with PyMOL 3.0.4. The custom Python scripts used for pore-volume voxel counting, contact occupancy accumulation, residue binning, and grid sampling are available from the corresponding author upon reasonable request.

## 3. Results and Discussion

This work investigates how pH-driven conformational changes at the AAV 5-fold pore govern the recognition and selective capture of genome-containing capsids by mixed-mode peptide ligands, and demonstrates that the structural dynamics of this conserved channel can be leveraged to tune affinity capture performance. To establish a mechanistic link from capsid topology to chromatographic selectivity, we designed a concerted experimental and computational approach that probes peptide–capsid interactions across a defined pH landscape and resolves their structural basis with atomic resolution. This study is articulated in four sections: we first established the physicochemical operating framework—namely, the ionic environment of the buffer systems (Section 3.1) and the pH-dependent ionization profiles of the three peptide ligands (Section 3.2). We subsequently traced how pH governs binding, enrichment of cell-transducing virions, and host cell protein clearance for AAV8 (Section 3.3) and AAV9 (Section 3.4), revealing serotype-specific patterns that point to fundamentally different modes of pH-responsive capsid–ligand engagement. We finally descended to the atomic scale with molecular dynamics simulations of the AAV8 and AAV9 5-fold pore under acidic, neutral, and basic conditions, showing how pH reshapes pore geometry, inter-residue contact topology, electrostatic surface potential, and solvent accessibility in serotype-conserved and serotype-divergent fashions (Section 3.5).

### 3.1. Buffer Selection and Ionic Strength

To isolate pH as the primary parameter governing peptide–capsid interactions, buffer systems were selected to span the target pH range while maintaining comparable ionic backgrounds across all conditions. Loading and equilibration buffers consisted of 10 mM sodium acetate at pH 5.0, 10 mM Bis-Tris at pH 7.0, and 10 mM Bis-Tris Propane at pH 9.0, each supplemented with 20 mM NaCl and 0.1% Pluronic F-68. Each buffer was chosen on the basis of its pKa proximity to the target pH, its established biological compatibility with AAV capsids, and its low intrinsic ionic strength; the latter ensured that NaCl would remain the dominant contributor to ionic strength, thereby maintaining comparable electrostatic conditions across all experiments. Acetate (pKa ~4.76) provides effective buffering capacity at pH 5.0 but, as a fully dissociating salt, contributes ~10 mM to the total ionic strength. Bis-Tris (pKa = 6.46) and Bis-Tris Propane (pKa_1_ = 6.8, pKa_2_ = 9.0) are zwitterionic buffers whose ionized fractions at pH 7.0 and pH 9.0 are small (2.2 mM and 5.1 mM, respectively), making NaCl the dominant contributor to ionic strength at these conditions and keeping total ionic strength within a narrow range of 22.2–25.1 mM, compared to 30.0 mM at pH 5.0 (Tables S1 and S2).

This modest but meaningful ~8 mM difference in total ionic strength between the acidic and near-neutral conditions has important mechanistic implications, as ionic strength governs peptide–capsid binding through two competing mechanisms. First, elevated ionic strength compresses the electrical double layer, weakening long-range electrostatic attractions between cationic peptide ligands and the net-negative capsid surface—particularly relevant above the isoelectric point of AAV8 and AAV9 (~pH 6.3), where the capsid carries an increasingly negative surface charge. This electrostatic shielding effect is most consequential for peptides TTFRAHHI and TYHHHHII, which rely heavily on charge-mediated contacts with anionic capsid residues. As a result, binding observed at pH 5.0—where total ionic strength is highest—may underestimate the true electrostatic contribution relative to equivalent low-ionic-strength conditions. Second, elevated salt concentrations can strengthen hydrophobic interactions through the salting-out effect, potentially amplifying the contribution of nonpolar residues such as Phe, Ile, and Val present across all three peptide sequences. The interplay between these opposing effects must therefore be considered when interpreting chromatographic adsorption data across pH conditions. We note that the contribution of this residual ionic strength difference was not deconvoluted experimentally: an ionic-strength-matched control, in which the pH 7.0 and pH 9.0 loading buffers are adjusted with NaCl to 30.0 mM total ionic strength, would be required to isolate it. Three features of the present dataset nonetheless indicate that it is a minor variable. The pH 9.0 condition, whose ionic strength lies within 3 mM of pH 7.0, produces opposite responses in different ligands, i.e., binding increases for TTFRAHHI and collapses for TYHHHHII, whereas electrostatic screening, being a property of the solution rather than of the ligand, would displace all three ligands in the same direction. At fixed pH 7.0, and therefore at identical ionic strength, AAV8 yields span 24.29% to 44.59% across the three ligands. And at pH 5.0, AAV9 retains measurable binding, while AAV8 does not, despite identical buffer conditions.

Elution was performed uniformly across all loading conditions using 0.2 M MgCl_2_ at pH 6.0. This condition disrupts peptide–capsid interactions through two synergistic mechanisms: the resulting ionic strength (~600 mM) triggers the dissociation of electrostatically bound capsids from the resin surface, while pH 6.0—near the His pKa ~6.2—promotes partial histidine deprotonation, progressively weakening the charge-mediated and coordinative contacts that anchor His-rich ligands to the AAV surface. Because the elution conditions were held constant across all experiments, differences in the recovered fractions can be attributed to binding and partitioning events at the loading pH rather than artifacts introduced by the elution conditions.

### 3.2. Peptide Net Charge as a Function of pH

The three peptide ligands display markedly different ionization profiles across the pH range tested, as calculated through iterative applications of the Henderson–Hasselbalch equation to all ionizable groups (Figure S1). Ligand RVVAVYRI contains two Arg residues, resulting in a persistently cationic character across the pH range examined. At pH 7, the peptide maintains a strong positive net charge due to the high pKa of Arg (~12.48), and this cationic character is largely retained even at pH 9. Consequently, electrostatic interactions are expected to play a dominant role in capsid binding, complemented by hydrophobic contributions from Val, Ala, Ile, and Tyr residues. Ligand TTFRAHHI carries an Arg residue (pKa ~12.48), which maintains a net cationic character across all pH values tested (i.e., net charges of +2.84, +1.11, and +0.09 at pH 5, 7, and 9, respectively). This permanent electrostatic anchor, combined with hydrophobic and π-π stacking contributions from Phe and directional contacts from two His residues that modulate their protonation state near neutral pH, confers the broadest operational pH window and the highest cross-condition robustness among the three ligands. Ligand TYHHHHII is the most pH-sensitive of the three, bearing four consecutive His residues that collectively produce the steepest charge transition across the series (i.e., the net charge shifts from +3.68 at pH 5 to −0.98 at pH 9). Near pH 7—approximately one unit above the imidazole pKa of ~6.2—the partial protonation state of the His cluster generates a precisely tuned electrostatic and hydrogen-bonding array that is uniquely complementary to the electrostatic landscape of genome-containing capsids—a selectivity mechanism that is entirely abolished at pH 9 upon complete histidine deprotonation.

### 3.3. AAV8: Mechanistic Interpretation of pH-Dependent Peptide Interactions

The pH-dependent binding profiles of the three peptide ligands RVVAVYRI, TTFRAHHI, and TYHHHHII toward AAV8 can be mechanistically rationalized by the interplay between peptide ionization state and the pH-responsive structural dynamics of the AAV8 capsid. At pH 5, the near-complete absence of productive AAV8 capture across all three resins (yield <5%) is consistent with two converging effects: first, the full protonation of the histidine residues (pKa ~6.2) present in the peptide sequences produces net cationic charges that are electrostatically unfavorable for engaging the AAV8 capsid; in turn the capsid surface undergoes a protonation-driven conformational rearrangement at acidic pH involving capsid residue H529, triggering the “pH quartet” transition (H529-E566-R392-Y707) and disrupting the icosahedral 2-fold interface; second, the associated disordering of the five radially oriented DE loops that constitute the 5-fold pore channel likely masks the native binding epitopes that are accessible at neutral pH. Among these two factors, the immediate suppression of binding at pH 5 is most plausibly dominated by unfavorable electrostatics, while DE-loop disordering further reduces access to the productive neutral-pH epitope.

At pH 7 and pH 9, where the capsid His residues are deprotonated and the native open-pore conformation is preserved, all three peptides regained binding activity, with the extent of capture being strongly modulated by their amino acid compositions. TTFRAHHI, bearing two histidine residues alongside a permanently cationic arginine (pKa ~12.5), achieved the highest capsid yields at both pH 7 (44.6%) and pH 9 (50.2%), a robustness attributable to the multimodal character of its interaction: the guanidinium group of arginine provides pH-independent electrostatic anchoring to anionic residues on the capsid surface, while the deprotonated imidazole rings of the histidine residues at pH 7–9 and the phenylalanine aromatic side chain enable complementary hydrophobic and π–π stacking contacts with solvent-exposed capsid residues. The single tyrosine in RVVAVYRI, embedded within a predominantly hydrophobic sequence (R, V, V, A, V, Y, R, I), likely contributes additional aromatic and hydrophobic contacts, while the two arginine residues maintain strong cationic character across the tested pH range, explaining the moderate yet consistent capsid recovery observed at both pH 7 (24.3%) and pH 9 (26.9%) (Figure 1 and Figures S2 and S3 and Table S3).

The most mechanistically informative ligand is TYHHHHII, whose four consecutive His residues, flanked by tyrosine and isoleucine, confer a unique pH responsiveness. At pH 7, namely ~1 unit above the His pKa of ~6.2, the near-threshold protonation state of these four His residues generates a partial cationic charge distribution that appears ideally suited to engage anionic or polar surface patches on the AAV8 capsid surface, yielding the highest transduction activity enrichment factor observed across the entire dataset (2.82). This selectivity for genome-containing and cell-transducing capsids is consistent with atomic force microscopy studies demonstrating that the surface charge and hydrophobicity differences between full and empty AAV8 capsids are significantly more pronounced at pH 7 than at pH 9 (Figure S1), suggesting that the electrostatic fingerprint distinguishing full from empty capsids is amplified at near-neutral pH. Critically, at pH 9, the complete deprotonation of all four His residues in TYHHHHII entirely abolished this interaction specificity: the AAV8 amount in the unbound fraction rose to 75.7% of the loaded virions, demonstrating that the activity-selective recognition mechanism depends on the precisely balanced partial protonation state of the poly-histidine motif achievable only near the pKa of His. Collectively, these results suggest that histidine-enriched peptides can function as pH-tunable molecular switches for AAV capture, with the number and spatial arrangement of His residues dictating both the pH window of productive binding and the degree of selectivity for cell-transducing virions. Such binding precision may offer a means for combining affinity capture with the enrichment of active virions in one chromatographic step.

### 3.4. AAV9: Distinct Serotype-Specific pH Response

The pH-dependent binding behavior of the three peptide ligands toward AAV9 recapitulates several serotype-conserved patterns observed with AAV8, while also revealing distinct features that reflect the unique structural dynamics of the AAV9 capsid across the pH range tested. At pH 5, unlike the near-complete absence of binding observed with AAV8, all three peptide resins captured marginal but non-negligible AAV9 fractions (yields 3.3–11.9%). This residual binding likely reflects subtle serotype-specific differences in the surface charge distribution of the capsid exterior. Specifically, the modest binding at pH 5 coincides with the reported AAV9 structural state in which the 5-fold channel becomes progressively occupied by a column-like internal density as the pH decreases from 7.4 toward 5.5, driven by a dual conformational switching of the conserved His229 residue near the interior of the 5-fold axis. The resulting partial closure of the pore—rather than full occlusion of binding-competent surface epitopes—may permit limited electrostatic contacts between the protonated imidazole rings of the His-containing peptides and residues on the still-exposed capsid surface within this pH range. Notably, the values of enrichment in transduction activity of the virions eluted at pH 5 were below 1.0 for all three peptides (0.20–0.51), confirming that while partial capsid binding occurs, it is non-selective and does not preferentially capture transcriptionally active, genome-containing capsids. This is consistent with the pH-driven dis-ordering of variable region II (VRII) of the DE loop at acidic pH that alters capsid surface presentation. In AAV9, the persistence of weak binding at pH 5 suggests that acidic-pH structural remodeling does not fully occlude the interface; rather, the principal limitation is loss of electrostatic compatibility, with residual exposure permitting only weak, non-selective adsorption.

At pH 7, where the AAV9 capsid adopts its native open-pore conformation and the 5-fold channel displays the intact column-like density characteristic of genome-packaged particles, the values of transduction activity enrichment rose sharply to 3.39, 3.21, and 5.61 for RVVAVYRI, TTFRAHHI, and TYHHHHII, respectively. These values are markedly higher than the equivalent AAV8 values at pH 7 (maximum 2.82 for TYHHHHII). This finding may reflect a serotype-specific feature of AAV9: genome loading generates a more pronounced column-like density at the 5-fold axis that distinctively remodels the pore surface at near-neutral pH, rendering it selectively accessible to the peptide ligands relative to empty capsids. At pH 7, TYHHHHII again achieved the highest activity enrichment (5.61-fold), reinforcing the interpretation that its four consecutive His residues, when partially protonated near the pKa of ~6.2, act as a pH-tunable electrostatic and hydrogen-bonding array that is specifically complementary to the surface architecture of full capsids. This selectivity was completely abolished at pH 9, as AAV9 binding was completely lost when all four His residues became fully deprotonated (Figure 2 and Figures S4 and S5 and Table S4).

At pH 9, TTFRAHHI demonstrated the highest and most robust capsid recovery (62.2% yield; 81.0% bound fraction), which was attributed to its arginine-anchored multimodal binding that confers operational robustness (Table S4). RVVAVYRI also showed a substantial yield improvement from pH 7 (6.6%) to pH 9 (28.3%), consistent with an increase in hydrophobic exposure of the capsid surface under alkaline conditions. The clearance of host cell proteins (HCP LRV) also improved for both RVVAVYRI and TTFRAHHI at higher pH, making pH 9 an ideal loading condition when yield maximization and HCP clearance are the primary process objectives for AAV9 purification with these ligands.

### 3.5. pH-Driven Remodeling of the 5-Fold Pore in AAV8 and AAV9: Inter-Subunit Contacts, Solvent Accessibility, and Electrostatic Landscape

The chromatographic results reported in Section 3.3 and Section 3.4 demonstrate that the three peptide ligands engage the AAV capsid surface in distinct, pH- and serotype-dependent fashions. The mechanistic origins of these binding and release patterns cannot be fully rationalized from the peptide sequence alone. To resolve the structural basis of these behaviors, we performed molecular dynamics (MD) simulations of the AAV8 and AAV9 5-fold pentameric assemblies at pH 5, 7, and 9. Our study enabled us to *(a)* trace how protonation-driven remodeling reshapes pore geometry, inter-subunit contact networks, solvent accessibility, and electrostatic surface potential in a serotype-specific manner, and *(b)* establish an atomic-scale framework for the mechanistic interpretation of the observed chromatographic selectivity.

#### 3.5.1. Pore Volume

**Figure 3:**
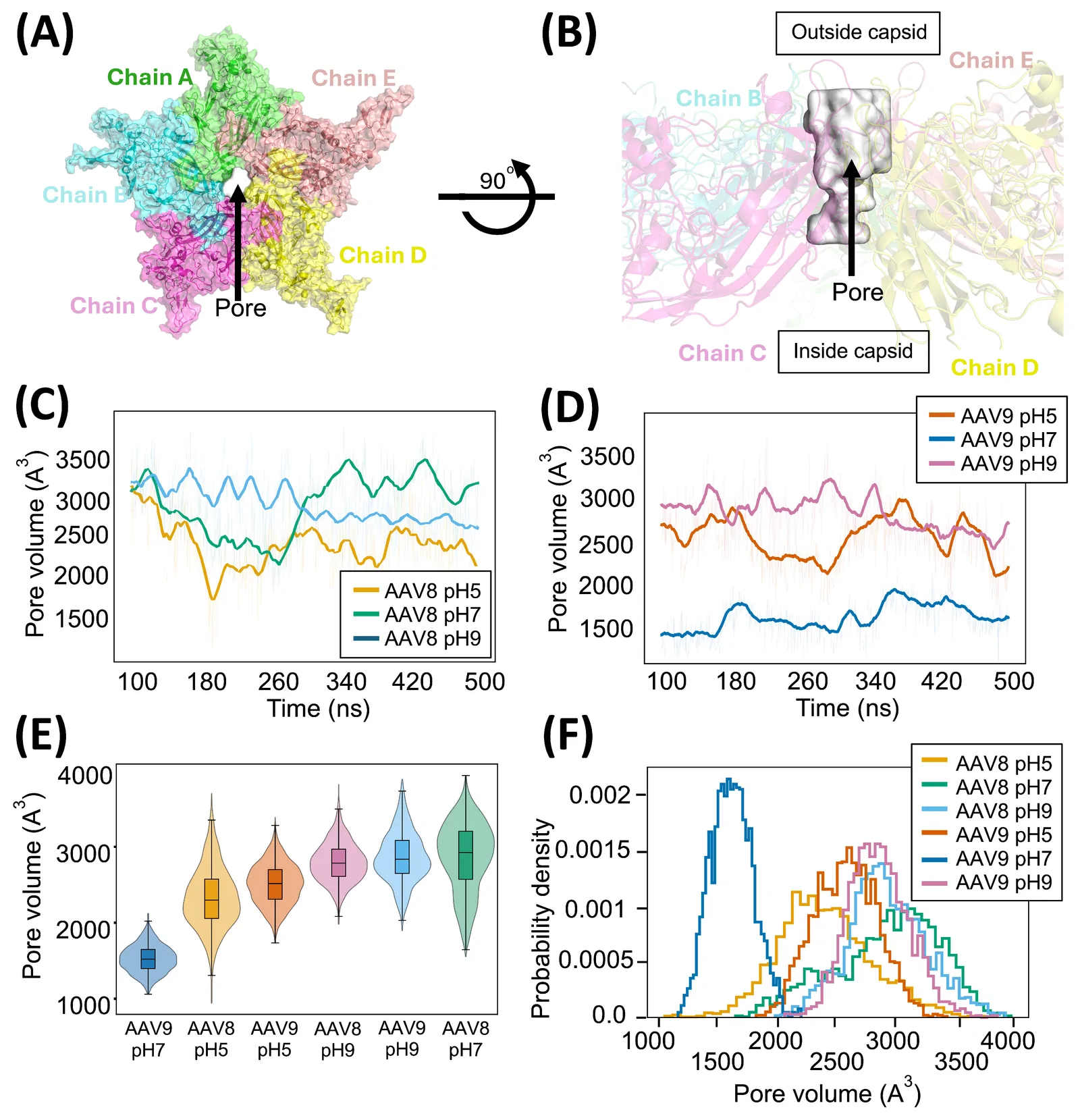
Pore volume as a function of pH. Panel (**A**), cartoon representation of the AAV 5-mer with each chain and pore labeled for ease of visualization. Panel (**B**), same cartoon representation as panel A, rotated 90 degrees, and surface representation turned off, each chain and pore labeled for ease of visualization. Panels (**C**,**D**), pore volume over the length of the simulation from 100 ns onward for AAV8 and AAV9, respectively. Panel (**E**), violin plots showing the distribution of pore volumes as a function of pH. Panel (**F**), probability density distributions (histograms) of pore volumes.

The pore-volume trajectories reveal distinct pH-dependent dynamics for each serotype. In AAV8, pore geometry is strongly modulated by pH and is characterized by a substantial conformational heterogeneity rather than uniformly stable fluctuations (Figure 3C,E,F). At pH 5, the pore samples a broad range of contracted conformations (~1680–3050 Å^3^; mean ~2374 Å^3^), consistent with a relatively collapsed but conformationally dynamic regime. At pH 7, the pore shifts to a larger-volume and a markedly more heterogeneous ensemble (~2125–3483 Å^3^; mean ~2897 Å^3^), consistent with the coexistence of two dominant conformational states. The two principal plateau states are centered at approximately 2490 Å3 and 3174 Å3, corresponding to a ~27% volumetric increase from the lower to the higher state. This indicates a substantial structural reorganization between the pore conformations. At pH 9, the pore remains in an expanded regime (~2600–3381 Å^3^; mean ~2910 Å^3^) but exhibits a lower variability relative to pH 7 (standard deviation ~212 Å^3^), suggesting a smoother free-energy landscape. Although a weak two-state structure can still be detected at pH 9—with the two levels centered near 2748 Å^3^ and 3139 Å^3^ (ΔV~14%)—these states are less well-resolved than at pH 7 and are more consistent with periodic pore “breathing” than with discrete state switching. Overall, AAV8 undergoes a pH-dependent transition from a contracted, dynamic ensemble at pH 5 to a strongly bimodal conformational landscape at pH 7, followed by a more continuously fluctuating expanded state at pH 9. Notably, the similar mean pore volumes at pH 7 and pH 9 belie clear differences in dynamics: pH 7 is dominated by switching between well-separated plateau states, whereas pH 9 exhibits reduced variance and weaker state separation.

AAV9 exhibits a markedly different and more ordered pH-dependent response (Figure 3D,F), consistent with the distinctive structural features of its 5-fold region described by crystallography [4]. At pH 7, the pore adopts a compact, highly stable conformation with consistently low volumes (~1400–1900 Å^3^; mean ~1607 Å^3^), indicative of a tightly closed state with minimal conformational variability; the pore-volume distribution is narrow, with minimal overlap with the ensembles sampled at pH 5 or pH 9 (Figure 3E,F). At pH 5, the pore expands to intermediate volumes (~2159–2956 Å^3^; mean ~2567 Å^3^), accompanied by moderate fluctuations but without clear evidence of discrete plateau states. At pH 9, the pore expands further (~2468–3194 Å^3^; mean ~2844 Å^3^), maintaining a relatively stable yet enlarged geometry. These observations indicate that AAV9 adopts its maximally constricted pore geometry at physiological pH, while deviations toward either acidic or basic conditions promote expansion, consistent with a biphasic response to pH. Nevertheless, the expanded states are asymmetric: the pore is consistently larger at pH 9 than at pH 5 (mean ~2844 Å^3^ vs. ~2567 Å^3^; ~11% increase), suggesting that alkaline conditions stabilize the most open pore conformation. Unlike AAV8, AAV9 remains dominated by a single conformational basin at each pH condition, with pH primarily governing the equilibrium pore geometry. The distributions shift systematically in position rather than in shape, in marked contrast to the breadth-modulated, multi-state behavior of AAV8. In summary, pH-dependent pore remodeling operates through fundamentally different mechanisms in the two serotypes: AAV8 responds through a flexibility-driven, multi-state mechanism in which protonation reshapes the breadth and topology of conformational sampling, whereas AAV9 behaves as a pH-gated switch, transitioning between compact and expanded pore states along a well-defined, low-heterogeneity trajectory. Taken together, these pore-volume behaviors help explain why pH 7 affords the strongest selective binding: in AAV8, a flexible bimodal pore likely generates transient binding-competent surface states, whereas in AAV9, the compact pH 7 pore defines a more discrete and selectively recognizable surface architecture.

These serotype-specific pore dynamics are consistent with the experimental observations presented in Section 3.3 and Section 3.4. The near-complete loss of AAV8 binding at pH 5 aligns with the contracted, conformationally disordered pore ensemble observed at that condition, which is likely to mask binding-competent surface sites. The residual AAV9 binding at pH 5, absent in AAV8, is consistent with AAV9’s intermediate pore volume at acidic pH, which allows partial surface accessibility even under unfavorable electrostatic conditions. Most importantly, the maximally constricted AAV9 pore at pH 7 and the bimodal AAV8 pore dynamics under the same condition coincide precisely with the pH at which both serotypes achieve their highest enrichment of cell-transducing virions, suggesting that pore geometry at pH 7 defines a topographically distinct binding surface that the partial protonation state of TYHHHHII is uniquely capable of exploiting.

#### 3.5.2. Contacts

**Figure 4:**
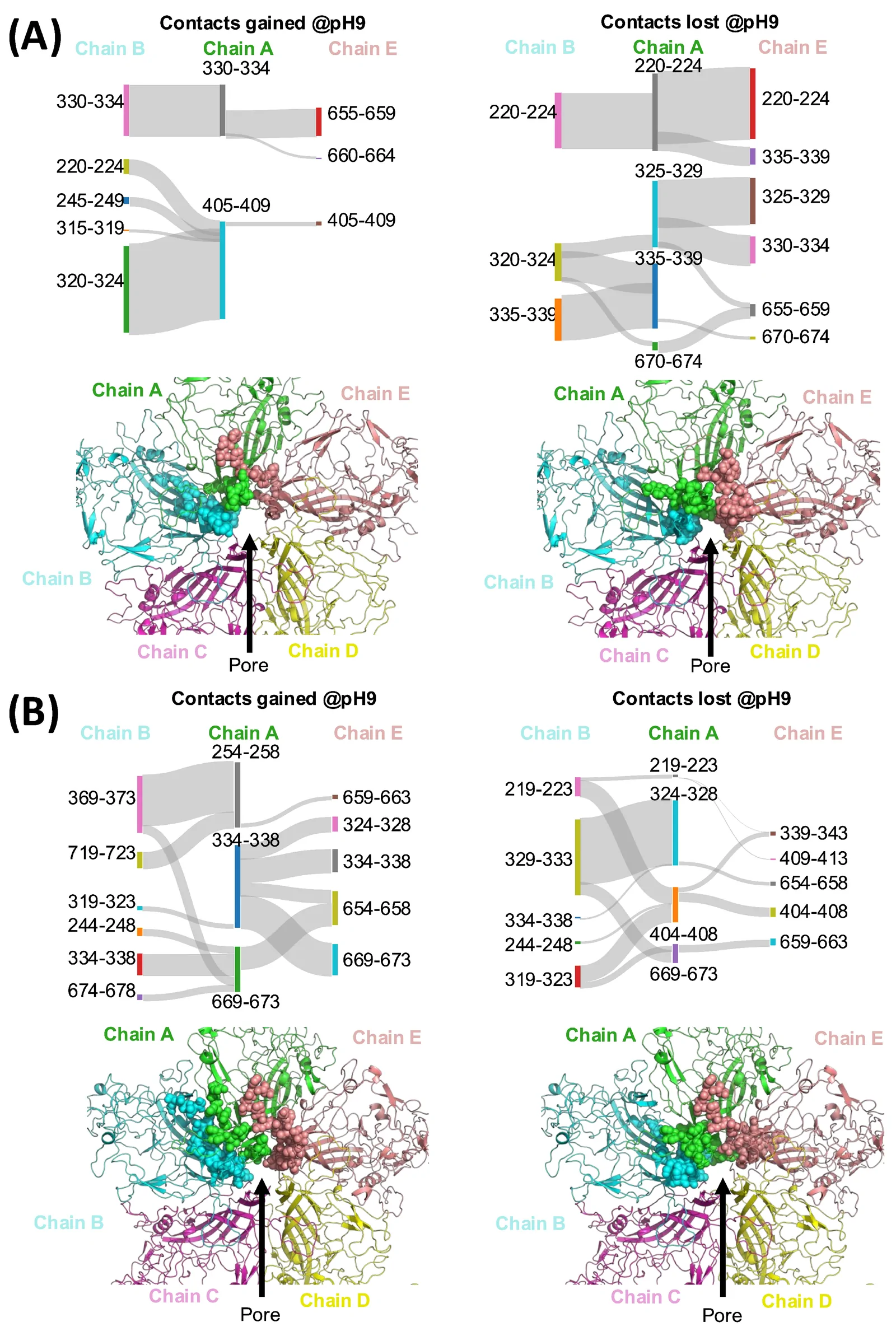
Dominant pH-dependent contact changes in AAV8 (**A**) and AAV9 (**B**) 5-mers. Top panels, Sankey plots summarizing the principal contact gains (left; pH 9 > pH 5) and losses (right; pH 5 > pH 9) centered on chain A interactions with neighboring chains B and E. Flow widths are proportional to the magnitude of the occupancy change and highlight the main chain A hubs through which pH-dependent contact remodeling is organized. Bottom panels, structural mapping of the dominant gained and lost regions onto the AAV8 and AAV9 5-mer assemblies. Residues participating in the dominant contact changes are shown as spheres. This figure is intended as a reduced, hub-focused summary of the major inter-subunit contact changes rather than an exhaustive view of the full contact network.

To characterize how inter-subunit interactions respond to changes in pH, inter-residue contact networks were quantified in 5-fold-symmetric assemblies of AAV8 and AAV9 at pH 5 and pH 9. Analysis focused on inter-subunit contacts between chain A and its neighboring subunits (chains B and E), enabling direct assessment of how structural perturbations propagate across adjacent interfaces within the capsid. Differences in contact occupancy were computed between the two conditions and visualized using centered Sankey diagrams to highlight coordinated changes across the B-A-E interaction axis (Figure 4; for clarity, only the dominant pH-dependent contact gains and losses and the principal chain A hubs through which these changes are organized).

The pH-dependent remodeling of interfacial contacts was evident in both serotypes, but AAV8 showed a comparatively modest and localized gain network. In AAV8, the total gain in contact occupancy was limited (ΣΔ ~2.66) and concentrated primarily in two chain A hubs: residues 330–334 at the pore summit and residues 405–409 on the luminal capsid surface (Figure 4A). The 330–334 hub gained contacts mainly with the same region on the adjacent chain and secondarily with residues 655–664, whereas the 405–409 hub gained contacts most strongly with residues 320–324 and more weakly with 220–224 and nearby luminal positions. Thus, the contact gains in AAV8 are best interpreted as localized rearrangements centered on a small number of pore-proximal and luminal hubs rather than as a broadly propagated inter-subunit rewiring event. In contrast, AAV9 displayed larger and more spatially distributed contact gains (ΣΔ ~5.97), consistent with a more coordinated pH-dependent reorganization of the interface. The dominant gain hubs were residues 334–338 at the pore, 669–673 on the adjacent exterior surface, and 254–258 at a more distal surface position (Figure 4B). Among these, residues 334–338 acted as the principal hub, gaining contacts with pore-adjacent residues 324–328 and 654–658, with additional coupling to 669–673. This organization defines a propagated interaction path extending from the pore toward neighboring surface loops, rather than a set of isolated local gains. Accordingly, the higher-pH contact gains in AAV9 reflect coordinated redistribution of inter-subunit connectivity across a broader structural region than observed in AAV8.

Contact losses driven by pH changes were even more structurally informative than contact gains. In both serotypes, contacts prevalent at pH 5 were selectively weakened or lost at pH 9 in an organized manner, and the total magnitude of these losses exceeded that of the gains. In AAV8, total contact loss (ΣΔ ~5.84) was concentrated in a contiguous pore-centered network involving chain A residues 220–224, 325–329, and 335–339. Residues 220–224 lost symmetric contacts with the same positions on adjacent chains and weaker contacts with 335–339, whereas residues 325–329 lost contacts with neighboring 325–329 and 330–334 positions and, to a lesser extent, with 320–324. Residues 335–339 likewise lost contacts with 320–324 and 335–339, forming a connected pathway that links the pore interior to adjacent surface loops. Thus, the AAV8 loss pattern is not a collection of isolated disruptions, but a cooperative weakening of a distributed pore-centered interaction network along the 5-fold axis. In AAV9, the loss signal was larger still (ΣΔ ~11.54), again exceeding the gain signal by about 2-fold. The dominant losses were centered on a smaller number of higher-weight residue clusters, particularly 219–223, 324–328, 404–408, and 669–673. Residues 219–223 lost symmetric contacts with the same positions on adjacent chains and with 339–343, whereas residues 324–328 lost contacts mainly with 329–333 and, secondarily, with 334–338 and 654–658. Residues 404–408 lost contacts with 219–223, 319–323, and neighboring 404–408 positions, while residues 669–673 lost contacts with 319–323 and 659–663. Together, these losses define a hub-dominated architecture in which 324–328 and 404–408 act as principal nodes linking pore-lining residues, adjacent surface loops, and more distal interior regions. Compared with AAV8, the AAV9 loss network is therefore more spatially constrained and dominated by fewer, stronger connections. Overall, in both serotypes, contact losses at alkaline pH exceeded gains, localized primarily to the 5-fold pore region, and tracked with the pore expansion described in Section 3.5.1. These results support a model in which acid-stabilized inter-subunit contacts help maintain pore integrity, whereas their disruption promotes pH-dependent opening. The topology of that disruption, however, remains serotype-specific: broader and more distributed in AAV8, but more hub-centered and localized in AAV9.

The asymmetry of gain vs. loss and its spatial localization at the 5-fold pore provide a structural rationale for the pH-dependent performance of the three peptide ligands. The coordinated collapse of pore-adjacent contacts at alkaline pH, along with the redistribution of interactions toward more distal surface regions, is consistent with the higher recovery of AAV8 and AAV9 obtained with TTFRAHHI at pH 9. The increased structural openness and redistribution of surface contacts at the 5-fold interface likely facilitate the multimodal engagement of Arg, His, and Phe side chains across a broader interaction surface. Conversely, the acid-stabilized pore-proximal contact network at pH 5, particularly the hub-centered loss topology in AAV9, explains the inability of all three peptides to engage the capsids at acidic pH. Indeed, a tightly cross-linked, low-accessibility pore surface cannot discriminate between full and empty capsids irrespective of the amino acid sequence of the peptide ligands.

The quantitative comparison of contact changes as a function of distance from the pore axis reveals a clear spatial bias in the remodeling process (Figure S6). In both serotypes, contacts lost at higher pH are preferentially located closer to the pore (~8–13 Å), whereas contacts gained are shifted toward more distal regions (~12–20 Å in AAV8; extending to ~24 Å in AAV9). This distance between loss- and gain-associated contact populations—with AAV8 showing an ~2–3 Å outward shift and AAV9 a smaller but consistent <1 Å shift (Figure S6A,B)—indicates that pH-dependent remodeling follows a directional pattern in which pore-proximal interactions are selectively weakened, while more distal contacts are redistributed or formed. This spatial pattern is further support-ed by time-resolved contact analyses (Figure S6C–F), showing that residue–residue contact fluctuations correlate with pore volume changes: contact variability in AAV8 is broadly distributed across pore volumes, whereas contacts in AAV9 cluster into more discrete regimes corresponding to compact and expanded pore states, demonstrating that contact remodeling is dynamically coupled to pore expansion. These contact-network differences provide a structural basis for the serotype-specific binding responses, with AAV8 undergoing more distributed local rearrangements and AAV9 exhibiting a more coordinated hub-centered reorganization that is consistent with its stronger pH-dependent ligand selectivity.

#### 3.5.3. Solvent Accessible Surface Area (SASA)

To quantify how pH modulates solvent accessibility across the 5-fold pore region of the capsid, residue-resolved SASA differences were computed for AAV8 and AAV9 (Figure S7). Across both serotypes, SASA differences exhibit a clear spatial organization, with distinct responses observed among pore-associated residues, luminal capsid surfaces, and exterior surfaces. In AAV8, SASA differences are moderate in magnitude and broadly distributed across the capsid. At pH 5, most residues show relatively small increases or decreases, typically within ±0.2–0.3 Å^2^, indicating limited structural rearrangement despite the global electrostatic shift. Notably, the residues in the pore-adjacent region (~220–340) show modest decreases in SASA (down to −0.3 Å^2^), consistent with partial pore compaction or increased solvent shielding at acidic pH. By contrast, at pH 9, these same regions exhibit increased solvent exposure (up to +0.3–0.4 Å^2^). However, these changes remain spatially diffuse, with both luminal and exterior residues showing mixed positive and negative contributions. This pattern is consistent with the distributed, heterogeneous reorganization of solvent accessibility previously inferred from AAV8’s multi-state, flexibility-driven pore dynamics.

In AAV9, SASA changes are larger in magnitude and more localized. At pH 5, luminal residues near the pore exhibit pronounced decreases in SASA (as low as −0.4 Å^2^), indicating a more tightly packed and solvent-shielded conformation relative to neutral pH conditions. At the same time, select exterior-facing regions show compensatory increases (+0.3–0.4 Å^2^), suggesting a redistribution of solvent exposure from the luminal capsid surface to the exterior. At pH 9, this trend reverses: pore-adjacent and luminal residues show substantial increases in SASA (up to ~0.4–0.5 Å^2^), while some exterior regions become more shielded. These pH-dependent exposure changes are spatially localized and concentrated within specific residue clusters, particularly those overlapping with the regions of contact loss—namely, residues 320–340 (pore-lining) and 650–680 (pore-adjacent exterior surface). Compared to AAV8, AAV9 displays a broader range of SASA differences (~0.8–1.0 Å^2^ total span vs. ~0.5–0.6 Å^2^ in AAV8) and a more spatially structured pattern.

These results establish solvent accessibility as an additional structural readout, coupled to pore dynamics and inter-subunit contact remodeling at the 5-fold interface. In AAV8, modest and spatially diffuse SASA differences are consistent with a flexible, multi-state ensemble in which no single structural element dominates solvent-exposure remodeling. In AAV9, larger yet spatially concentrated SASA changes co-localize with regions of inter-subunit contact loss, with pore-adjacent residues simultaneously exhibiting enhanced solvent exposure. Thus, pH-dependent changes in solvent accessibility do not uniformly expose or bury the capsid surface, but instead regulate the formation of localized binding-competent patches that likely contribute directly to differences in peptide capture and enrichment of cell-transducing virions. This is consistent with the gate-like, hub-centered transition described in Section 3.5.1 and Section 3.5.2, in which focused pore opening drives a coordinated redistribution of surface accessibility across a small number of structurally critical residue clusters.

#### 3.5.4. Electrostatics

**Figure 5:**
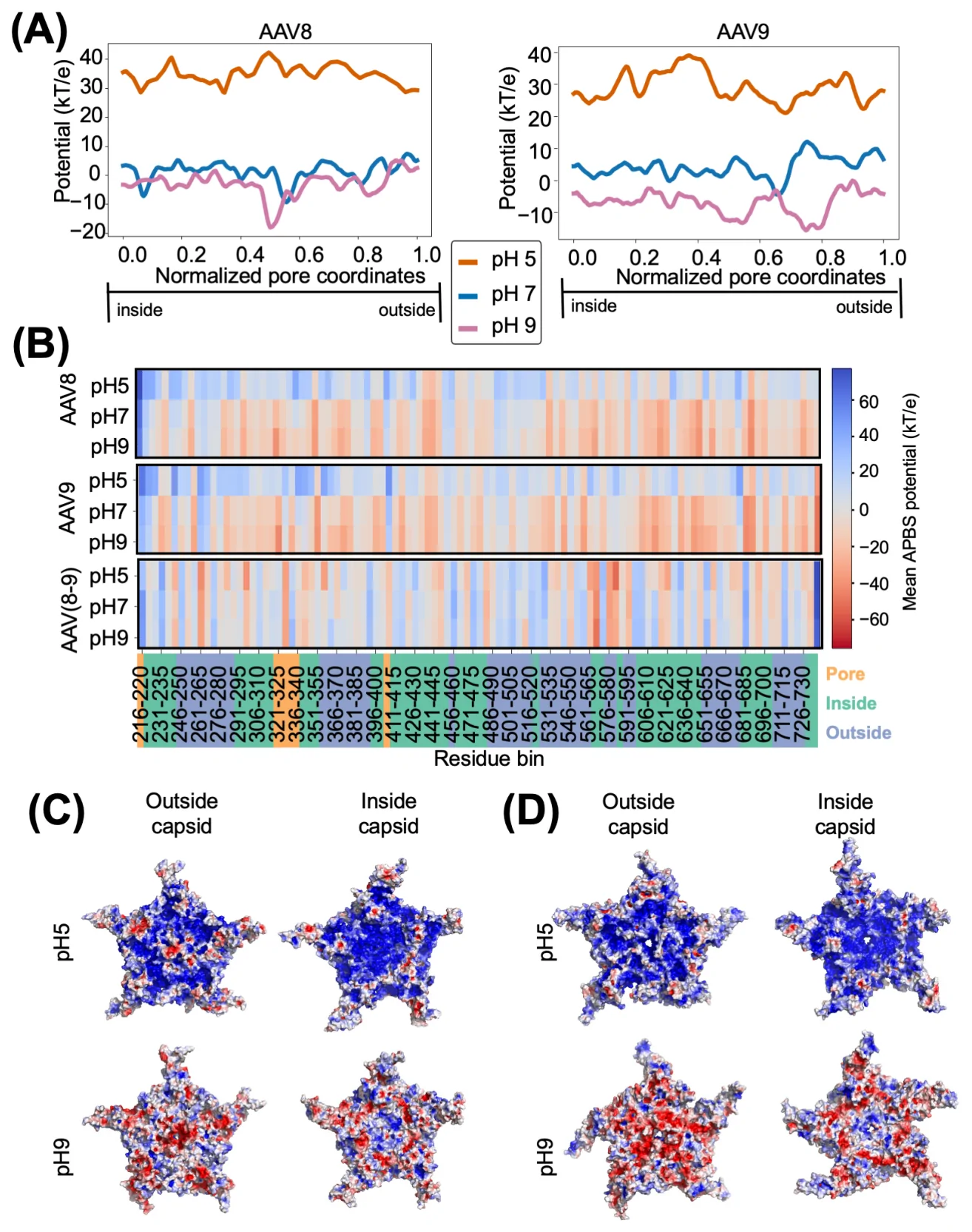
Comparison of the electrostatic properties of AAV8 and AAV9 as a function of pH. (**A**) Mean electrostatic potential (kT/e) along the normalized 5-fold pore coordinate for AAV8 (**left**) and AAV9 (**right**). The pore coordinate was defined identically for both serotypes, with 0 corresponding to the lumen-facing/internal end of the pore and 1 corresponding to the exterior-facing/pore-summit end. Line colors are consistent across both serotypes: pH 5, orange; pH 7, blue; pH 9, green. (**B**) Residue-binned mean APBS electrostatic potentials for AAV8 (**top**), AAV9 (**middle**), and AAV8-AAV9 (**bottom**). Rows correspond to pH 5, pH 7, and pH 9. Blue indicates positive potential and red indicates negative potential. Within each panel the three pH rows share a common color scale, set by the maximum absolute potential of that panel, so that pH conditions may be compared directly. The scales of the three panels are normalized independently; magnitudes should therefore not be compared between panels, and in particular between serotypes. Residue bins are annotated by structural region as pore, inside, or outside. (**C**,**D**) Surface electrostatic maps of AAV8 (**C**) and AAV9 (**D**) at pH 5 and pH 9, shown from the outside and inside capsid views.

To investigate how pH modulates the electrostatic landscape of the AAV capsids, electrostatic potentials were computed and compared for AAV8 and AAV9 at pH 5, 7, and 9 (Figure 5). These analyses reveal a pH-dependent shift in electrostatic potential across serotypes, accompanied by spatially localized and serotype-specific deviations that are mechanistically informative. The potentials sampled along pore-defining residues show a consistent pH-dependent trend for both serotypes (Figure 5A): the capsid pore carries strongly positive potentials at pH 5, near-neutral values at pH 7, and increasingly negative potentials at pH 9. In AAV8, pore potentials at pH 5 are predominantly positive, spanning approximately +25 to +40 kT/e with a mean near ~32–35 kT/e; at pH 7 the values collapse toward near-neutral levels, spanning ~0 to +10 kT/e; finally, at pH 9, the potential shifts further negative, with values ranging between −5 and −20 kT/e, indicating a full reversal of electrostatic character across the pore axis. In contrast, AAV9 exhibits a much broader dynamic range across all conditions. At pH 5, pore potentials reach similar or slightly higher maxima (+30 to +40 kT/e), but display greater variability along the pore axis. At pH 7, AAV9 potentials are more dispersed than those of AAV8, spanning from −5 to +15 kT/e, indicating greater local heterogeneity even near neutral pH. At pH 9, AAV9 exhibits the most negative potentials of any condition tested, ranging from −10 to −25 kT/e, with more abrupt spatial fluctuations than AAV8. This corresponds to a dynamic range of the pore of ~60 kT/e in AAV9 (from strongly positive at pH 5 to strongly negative at pH 9), compared to a narrower ~50 kT/e in AAV8. AAV9 also displays greater local deviations along the pore axis. This pronounced electrostatic sensitivity is consistent with experimental reports that the aggregation, self-interaction, and genome-release behaviors of AAV9 capsids are strongly governed by surface electrostatics [29]. Therefore, while both serotypes undergo a qualitatively similar electrostatic transition with pH, AAV9 exhibits both greater magnitude and higher spatial variability, whereas AAV8 maintains a smoother, more uniformly distributed electrostatic profile along the pore.

Averaging the electrostatic potentials across defined structural regions—namely, the capsid lumen, the pore, and the external surface—reveals that this global shift is accompanied by consistent region-dependent offsets (Figure S8A). In AAV8, the mean potentials decrease monotonically with increasing pH across all regions (lumen ~50, 35, 25 kT/e; pore ~45, 30, 25 kT/e; outside ~40, 25, 20 kT/e; at pH 5, 7, 9), indicating a largely homogeneous and spatially undifferentiated electrostatic response. In contrast, AAV9 shows both higher absolute potentials and stronger regional differentiation (lumen: 55, 30, 20 kT/e; pore ~45, 25, 22 kT/e; outside ~40, 25, 20 kT/e; at pH 5, 7, 9), with the most pronounced inter-regional gradients occurring within the capsid lumen. These data indicate that AAV9 exhibits not only a larger overall electrostatic response, but also a steeper spatial gradient between luminal and exterior. The comparison of AAV8 and AAV9 electrostatic profiles along the normalized pore axis shows localized deviations of up to ±10–15 kT/e, particularly at pH 5 and pH 9 (Figure S8B; *note:* within each panel, the three pH rows share a common color scale, so that pH conditions can be compared directly; the panels are normalized independently, and magnitudes should therefore not be compared between panels.). These differences are not uniform along the pore axis but arise at discrete positions, indicating that the electrostatic differences between serotypes are spatially structured rather than globally offset.

Residue-resolved electrostatic differences further highlight pH-responsive regions (Figure 5B). In AAV8, the largest positive deviations at acidic pH (pH 5–7) are concentrated in residue bins 286–290 (+41.3 kT/e; exterior surface, distal to the pore), 361–365 (+36.0 kT/e; exterior surface, pore-proximal), and 336–340 (+33.9 kT/e; pore-lining residues), whereas the largest negative deviations occur in residue bins 231–235 (luminal residues, distal to the pore), 611–615 (distal to the pore), and 371–375 (+27–31 kT/e; distal to the pore, but closer). These changes are distributed across both luminal and exterior regions, consistent with the relatively diffuse electrostatic reorganization characteristic of AAV8. At higher pH (pH 7–9), AAV8 exhibits weaker and more spatially restricted changes, with the largest negative shifts localized near the pore-lining region including residue bins 336–340 (−19.6 kT/e; pore-lining) and 321–325 (−17.3 kT/e; pore-lining), indicating an electrostatic destabilization localized at the 5-fold axis. In contrast, AAV9 shows substantially larger and more spatially concentrated electrostatic shifts. At low pH (pH 5–7), the strongest positive deviations are observed in residue bins 611–615 (+43.9 kT/e; distal to the pore), 356–360 (+43.8 kT/e; distal to the pore), and 281–285 (+43.6 kT/e; pore-proximal exterior), indicating a substantially larger response relative to AAV8. At higher pH (pH 7–9), the largest negative shifts localize near pore-critical regions, including residues 336–340 (−18.5 kT/e; pore-lining), 321–325 (−16.0 kT/e; pore-lining), and 366–370 (−15.4 kT/e; pore-proximal exterior). Notably, these residue clusters overlap with regions implicated in contact loss and pore-adjacent remodeling in Section 3.5.2, suggesting a coupling between electrostatic redistribution and structural rearrangements described above.

To isolate the residues most responsive to pH, the top 10% of electrostatic changes were extracted and analyzed separately. In AAV8 (Figure S8C), the largest deviations at acidic pH (pH 5–7) are distributed across both the exterior capsid surface and the pore region, with peak shifts reaching ~+35–40 kT/e in residue bins such as 286–290 (exterior surface residues) and 336–340 (pore-defining residues). This distribution indicates that electrostatic amplification in AAV8 at low pH is not confined to a single structural region but encompasses both the external surface features and the 5-fold pore. In contrast, the largest negative deviations at higher pH (pH 7–9) are more spatially restricted and preferentially confined to pore-adjacent residues (e.g., 321–340; pore-lining), with magnitudes of −15 to −20 kT/e, pointing to targeted electrostatic destabilization at the 5-fold interface. In AAV9, the top 10% electrostatic changes are both larger in magnitude and more spatially organized (Figure S8D). At lower pH, the strongest positive shifts reach ~+40–45 kT/e and are concentrated in luminal-facing regions—namely, residues 281–285, 356–360, and 611–615—indicating a highly amplified and spatially coherent electrostatic response. At higher pH, the largest negative deviations (−15 to −20 kT/e) again localize near pore-adjacent residues (321–340), mirroring the regions of maximal contact loss and pore expansion identified in Section 3.5.1 and Section 3.5.2. Compared to AAV8, the top-ranked residues in AAV9 are fewer in number but contribute disproportionately to the overall electrostatic shift, indicating a more concentrated and structurally coupled redistribution of surface charge. These results demonstrate that, while AAV8 distributes electrostatic changes across a broader set of residues, AAV9 concentrates its largest shifts in a small number of pore-adjacent structural clusters, consistent with the hub-centered remodeling topology established in Section 3.5.2.

The presentation of the electrostatic potential landscape on the capsid surface in Figure 5C,D reinforces these observations. At pH 5, both serotypes exhibit predominantly positive electrostatic character across the luminal and exterior surfaces. However, AAV9 displays a more extensive and contiguous regions of positive potential. At pH 9, both systems shift toward negative potential, although AAV9 presents more spatially coherent and extensive anionic patches, particularly on the luminal capsid surface; conversely, AAV8 retains a more heterogeneous pattern with interspersed electrostatic regions distributed across both surfaces. These patterns are consistent with the structural behavior characterized in Section 3.5.1, Section 3.5.2 and Section 3.5.3: in AAV8, the heterogeneous electrostatic redistribution is consistent with its multi-state, flexibility-driven pore dynamics and broadly distributed contact network; in AAV9, the larger magnitude and spatially localized electrostatic shifts, particularly near pore-adjacent residue clusters, correlate with the hub-centered contact loss and gate-like pore expansion described above. These findings support a hierarchical mechanistic model in which pH-dependent protonation events drive electrostatic redistribution at the 5-fold pore, which reshapes inter-subunit contact networks and governs pore expansion and conformational dynamics in a serotype-specific manner. Accordingly, the electrostatic data link pore remodeling to chromatographic behavior by showing that pH 7 creates the most favorable charge environment for partial histidine-mediated recognition, whereas pH 5 and pH 9 shift the interface toward less productive binding states.

The electrostatic landscape emerging from these analyses is consistent with the pH-dependent chromatographic binding profiles reported in Section 3.3 and Section 3.4. The strongly positive potentials at pH 5, where both the His residues of the peptide ligands and the protonatable residues of the capsid carry net positive charges, are consistent with the near-null binding observed for both AAV8 and AAV9 at acidic pH, as the electrostatic repulsion between peptide and capsid surface prevents adsorption. At pH 7, the decrease of pore potentials toward near-neutral values in both serotypes creates an electrostatic environment complementary to the His cluster of TYHHHHII, whose milder net charge can engage the anionic patches on the capsid identified in the residue-resolved analysis. The shift toward strongly negative pore potentials at pH 9 (more pronounced in AAV9) is consistent with the loss of TYHHHHII binding, as full His deprotonation produces a neutral-to-negative peptide, which repels an increasingly anionic capsid surface. Conversely, the pH-independent cationic character of Arg in TTFRAHHI allows effective binding across the pH 7–9 range despite this progressive electrostatic shift, explaining its broad operational window.

### 3.6. Potential Peptide Binding Interface

**Figure 6:**
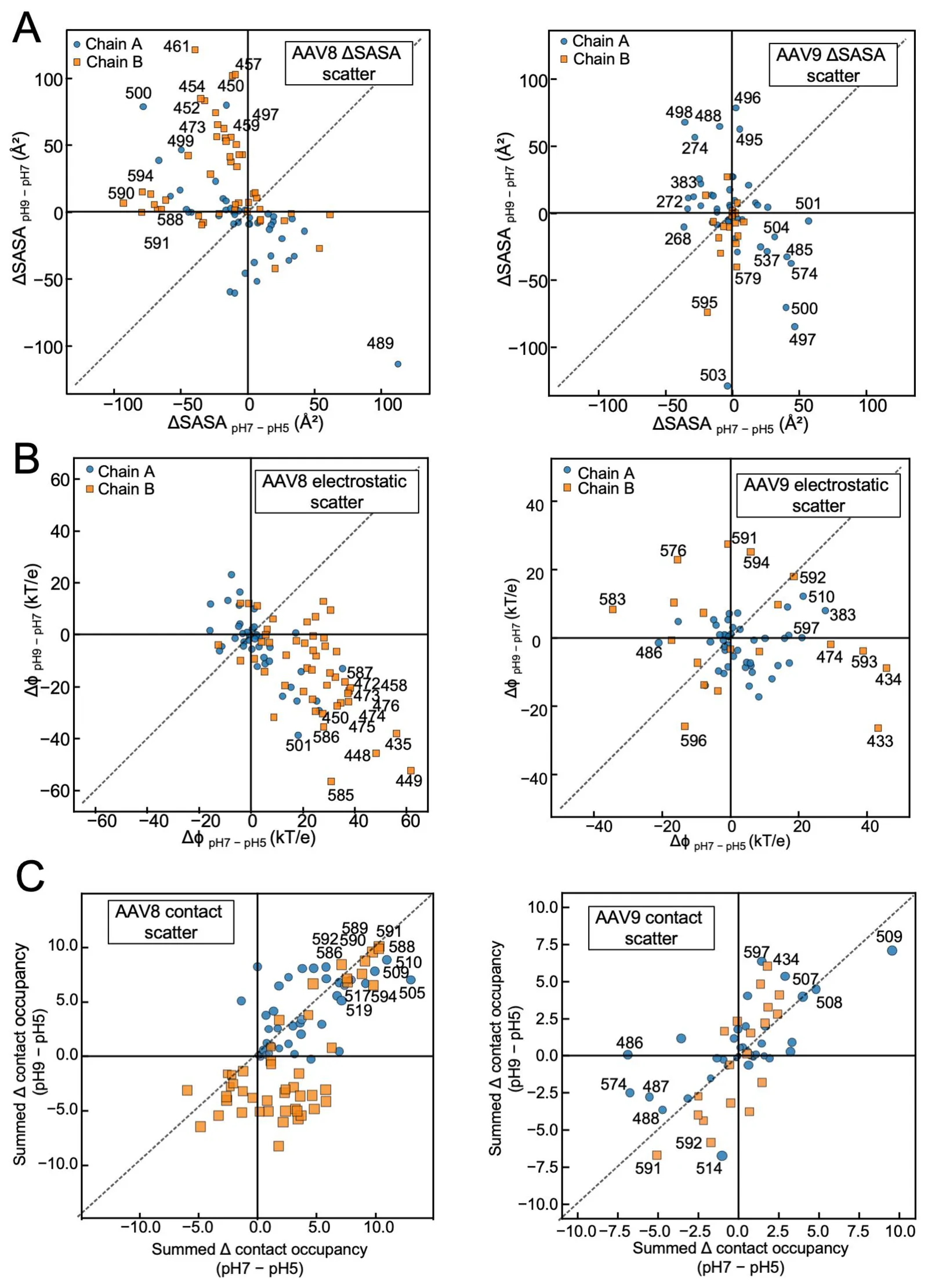
Comparison of residue-level remodeling across sequential pH transitions within the peptide-binding surfaces of AAV8 and AAV9. (**A**) Scatter plots comparing residue-level solvent accessible surface area changes (ΔSASA) between the pH7–pH5 transition (*x*-axis) and the pH9–pH7 transition (*y*-axis) for AAV8 (**left**) and AAV9 (**right**). (**B**) Scatter plots comparing residue-level electrostatic potential changes (Δϕ) across the same sequential pH transitions. (**C**) Scatter plots comparing summed residue-level contact occupancy remodeling between the pH transitions. Values represent the summed occupancy change for all residue–residue contacts involving each binding-site residue. Each point represents an individual binding-site residue from chain A (blue circles) or neighboring interfacial chains (orange squares). The dashed diagonal indicates equal remodeling magnitude across both transitions. Residues positioned along the diagonal undergo similar directional changes across both pH transitions, whereas residues displaced from the diagonal exhibit non-linear behavior.

The chromatographic data presented in Section 3.3 and Section 3.4 show that TYHHHHII achieves activity-based selectivity only at pH 7, that TTFRAHHI maintains broad-pH recovery owing to its Arg-driven multimodal binding, and that RVVAVYRI shows a stable, non-selective hydrophobic binding mode. To resolve why the three peptides, which differ mostly in their ionizable residue content, interact with the same capsid surface in such distinct ways, we characterized the structural, electrostatic, and contact properties of the putative peptide-binding interface on the AAV capsid surface as a function of pH. Within the putative binding epitope, pH-dependent remodeling was concentrated in chain A and the adjacent interchain interface (Figure 6 and Figure S9A) [10,13,30]. In both serotypes, residues within the chain A binding surface and adjacent interchain interface showed large pH-dependent changes in solvent exposure. These changes were spatially focused in localized hotspots rather than uniformly distributed across the surface, indicating that the peptide-binding region populates multiple solvent-exposure states rather than undergoing a simple open/closed transition.

The comparison of the pH transitions further clarifies the various remodeling behaviors between serotypes (Figure 6A). In AAV8, residues with high-magnitude SASA changes present a negative ΔSASA (solvent burial) at pH 5 to 7 and a positive ΔSASA (re-exposure) at pH 7 to 9. Chain A residues exhibiting the largest changes include Gln489 (+112.5, −114.1 Å^2^), Asn500 (−78.9, +78.9 Å^2^), and Ser501 (−67.1, +38.6 Å^2^), while the adjacent interchain interface contributes prominent hotspots at Arg450 (−11.9, +102.1 Å^2^), Thr457 (−9.5, +103.0 Å^2^), and Gln461 (−39.8, +121.8 Å^2^). The distribution of responsive residues across both chain A and the neighboring interchain interface indicates that SASA remodeling in AAV8 is structurally coupled across multiple subunit elements. In contrast, AAV9 exhibits a more spatially constrained response dominated by chain A residues—including Asn497 (+46.9, −84.9 Å^2^), Asn498 (−35.2, +67.8 Å^2^), and Trp503 (−3.3, −129.2 Å^2^)—with comparatively smaller yet still meaningful interfacial contributions from residues such as Gln474 (−8.6, −29.9 Å^2^), Gln579 (+3.6, −40.2 Å^2^), and Trp595 (−18.5, −74.0 Å^2^). In AAV8 the pH-dependent remodeling is broadly amplified across the interface, while in AAV9 the response is concentrated in specific aromatic and polar hotspots (Trp503 and Trp595), indicating that pH-dependent SASA remodeling is more strongly localized within the primary chain A binding surface rather than propagating across the interchain network. Despite these shared hotspot regions, the remodeling topology differs between serotypes: AAV8 distributes the response more broadly across chain A and the interchain interface, whereas AAV9 concentrates it more strongly within the primary chain A surface. At the epitope level, AAV8 shows broader interchain coupling, whereas AAV9 shows more localized chain A-centered remodeling.

Within the putative binding epitope, electrostatic potential was redistributed in a strongly pH-dependent manner [13,17,31] (Figure 6B and Figure S9A). In both serotypes, numerous residues exhibited coordinated changes in mean electrostatic potential concentrated in localized hotspots rather than through uniform polarization of the interface (Figure 6B). Notably, this behavior was more pronounced in AAV8, which exhibited a large increase in mean site potential during the pH 5–7 transition (Δϕ_5–7_ = +13.42 kT/e; pH 5 = 3.33, pH 7 = 16.75) followed by substantial reversal at pH 9 (Δϕ_7–9_ = –8.98 kT/e; pH 9 = 7.77); the hotspots driving these transitions include interfacial residues Leu448, Ser449, and Arg450, and the 585–587 region (largest positive Δϕ_5–7_), as well as Asn475, Ser501, Arg435, and Leu586 (largest negative Δϕ_7–9_). In contrast, AAV9 displayed a more modest electrostatic response, with a smaller increase from pH 5 to 7 (Δϕ_5–7_ = +3.63 kT/e; pH 5 = 1.35, pH 7 = 4.99) and limited reversal at pH 9 (Δϕ_7–9_ = −1.20 kT/e; pH 9 = 3.79). The responsive residues are confined to the interchain interface—chiefly, Ala510, Ala591, Gln592, and Gly594 (largest positive Δϕ_5–7_) and Asp433, Arg434, Gln474, and Thr593 (largest negative Δϕ_7–9_). Their lower-amplitude response is consistent with serotype-specific sequence differences that reduce local electrostatic amplification at the AAV9 epitope.

Residue-level contact analysis showed that the putative binding epitope remodels differently in AAV8 and AAV9 (Figure 6C and Figure S9B). In AAV8, contact remodeling is characterized by large-amplitude changes across both chain A and the interchain interface (ΔContacts_5–7_, ΔContacts_7–9_). The interfacial residues define a prominent hotspot centered in the 586–593 region—chiefly, Gln589 (+12.6, +12.2), Asn590 (+11.9, +9.6), Thr591 (+12.7, +10.6), Ala592 (+11.5, +8.8), and Pro593 (+12.0, +9.7)—where contacts gained at pH 7 are largely retained or further stabilized at pH 9. Within chain A, large-amplitude gains were observed for Trp505 (+13.0, +7.0), Lys510 (+10.2, +7.2), and Leu519 (+11.0, +8.9), which correspond to key positions within the peptide-binding surface, with a partial reversal at pH 9 for several intra-chain positions (Figure 6C). Electrostatic-contact coupling was also evident in AAV8. During the pH 5 to 7 transition, interfacial residues including Arg435, Ser449, Gln587, Asn590, Thr591, Ala592, and Pro593 shifted toward higher contact occupancy and more positive electrostatic potential, whereas the pH 7 to 9 transition drove residues such as Leu448, Ser449, Arg450, Glu452, and Asn500 toward reduced contact occupancy and a more negative electrostatic regime (Figure S9B). A subset of residues, including Gln489, retains positive electrostatic character despite continued contact gain, indicating that the pH 9 state represents a partially reorganized, rather than simply reversed, interaction ensemble. In contrast, AAV9 displays a more residue-specific and spatially constrained pattern. Chain A contact gains are concentrated at Trp503 (+0.4, +9.1), Ala506 (+4.3, +7.8), Ser507 (+5.6, +4.6), and Trp509 (+9.6, +7.1), while prominent losses occur at Gln486 (−6.9, +0.1), Thr574 (−12.0, −5.1), and Arg514 (−1.0, −6.7). The interchain contributions are more limited, with Arg434 (+1.8, +6.0) and Ala591 (−5.1, −6.7) being the most notable interfacial positions (Figure 6C). The electrostatic contact coupling in AAV9 does not have the directional coherence noticed in AAV8. During the pH 5 to 7 transition, most residues cluster near the origin with modest changes, although Asp433, Arg434, Thr574, and Thr593 exhibit strong electrostatic responses despite limited contact reorganization (Figure S9B, bottom-left). During the pH 7 to 9 transition, residues are distributed across multiple response regimes: Thr574 shows positive electrostatic shifts with modest contact gain, while Asn496, Trp503, and Ala506 undergo substantial contact redistribution accompanied by increasingly negative electrostatic potentials (Figure S9B, bottom-right). Although both serotypes remodel analogous regions of the epitope, AAV8 does so more broadly across the interface, whereas AAV9 concentrates the response into fewer local hotspots.

A question raised by the chromatographic performance of TYHHHHII focuses on its selective capture of genome-containing over empty capsids at pH 7, resulting in enrichment factors of 2.82 (AAV8) and 5.61 (AAV9). Atomic force microscopy studies have demonstrated that full and empty AAV capsids differ in surface stiffness, charge density, and hydrophobicity, with these differences being more pronounced at near-neutral pH [32]. HDX-MS studies have shown that genome encapsidation stabilizes specific surface loops and reduces deuterium exchange at regions overlapping with the pH-responsive hotspots identified here (i.e., the 497–505 and 586–593 regions of the chain A binding surface) [33], indicating that the transgene presence rigidifies the outer capsid surface in a manner that alters the solvent-accessibility and the contact-remodeling profile of the binding interface [26,31]. We therefore propose that at pH 7, the partial protonation of TYHHHHII generates a precisely tuned electrostatic and hydrogen-bonding array that is complementary to the remodeled binding epitope of full capsids, with this complementarity being more sharply defined in AAV9 than in AAV8. This more spatially focused remodeling of the chain A/interchain epitope in AAV9 provides a plausible structural basis for the higher activity-based selectivity of TYHHHHII in AAV9 relative to AAV8. This selectivity is lost at pH 9 because the His deprotonation eliminates the charge-mediated contacts required to discriminate between the subtly different surfaces of full and empty particles. We emphasize that this account is a mechanistically motivated hypothesis rather than a demonstrated mechanism. The quantity measured here is the transduction activity of the eluate relative to the load (Equation (2)); no direct determination of the full-to-empty capsid ratio was performed on the chromatographic fractions, and the molecular dynamics systems are capsid assemblies without a packaged genome. The proposition that partial protonation of TYHHHHII discriminates between the surface states of genome-containing and genome-free particles is therefore testable, and would be tested directly by chromatography on separately purified full and empty populations, by analytical ultracentrifugation, charge-detection mass spectrometry, or genome-to-capsid ratio determination on each fraction, and by molecular dynamics of genome-containing assemblies.

In summary, the SASA, electrostatic, and contact analyses presented above converge on a unified model in which peptide recognition is governed by a conserved, pH-responsive binding interface that undergoes coordinated, multidimensional remodeling rather than adopting a single rigid binding conformation. The adaptation of the capsid surface of each serotype to pH occurs through serotype-specific mechanisms: AAV8 implements a broadly distributed and highly coupled remodeling network spanning chain A and the surrounding interchain interface, whereas AAV9 relies on spatially focused, chain A-centered rearrangements with weaker interchain coupling. The conservation of pH-responsive hotspots across both serotypes supports our experimental findings that serotype-agnostic peptide ligands can be designed by targeting the shared dynamic interface. At the same time, the distinct remodeling topologies provide a rational basis for the rational engineering of serotype-selective ligands that preferentially stabilize the localized electrostatic and structural states unique to each capsid, thereby establishing a mechanistic bridge between empirical peptide discovery and the de novo design of programmable ligands for AAV capture and purification. These results suggest several practical sequence-design rules for AAV affinity ligands. They derive from three mechanistically contrasting ligands selected from a larger ensemble screened in a separate ligand-design study [17], and are therefore to be read as mechanistically motivated guidelines rather than as relationships established by systematic screening within the present work. First, clustered histidines are advantageous when activity-based selectivity is desired near neutral pH, because partial His protonation can create a tunable electrostatic and hydrogen-bonding array that discriminates among closely related capsid surface states, as exemplified by TYHHHHII. Second, inclusion of at least one permanently cationic anchor residue such as Arg favors robust binding across a broader pH window, as observed for TTFRAHHI. Third, aromatic and hydrophobic residues such as Tyr, Phe, Ile, and Val should be used to reinforce binding through non-electrostatic contacts once initial recognition has occurred. Finally, the spacing and local context of these residues should be tuned to the serotype-specific remodeling of the chain A/interchain epitope rather than assuming a universal AAV binding motif.

## 4. Conclusions

This study presents a detailed pH-resolved structural and physicochemical comparison of the 5-fold pore in AAV8 and AAV9, integrating molecular dynamics simulations with chromatography experiments using three mixed-mode peptide ligands. The central finding is that the pH-driven remodeling of the 5-fold pore—encompassing inter-subunit contact networks, solvent accessibility, and electrostatic potential—is not merely a passive consequence of protonation chemistry but is a structurally organized, serotype-specific gating mechanism that governs the accessibility and character of the ligand-binding interface on the capsid. Despite sharing 83% capsid protein sequence identity, AAV8 and AAV9 implement this gating mechanism through fundamentally different topologies: AAV8 responds to pH through a broadly distributed, flexibility-driven multi-state ensemble, whereas AAV9 undergoes discrete, hub-centered transitions between compact and expanded pore states. These differences are reflected in distinct electrostatic and solvent-accessibility profiles that define a conserved yet serotype-tunable ligand-binding surface, providing the first mechanistic explanation for why mixed-mode ligands can engage both serotypes while providing different purification performance.

On the chromatographic side, the three peptide ligands evaluated span a functional spectrum that maps directly onto this structural framework. TTFRAHHI, featuring a pH-independent arginine residue and reinforced by multimodal histidine and hydrophobic contacts, delivered the highest capsid recovery across both serotypes and the broadest operational pH window, emerging as the preferred candidate where yield and cleaning-in-place compatibility are primary objectives. TYHHHHII demonstrated that a tetra-histidine motif partially protonated near neutral pH functions as a programmable molecular switch, selectively enriching genome-containing over empty capsids with activity enrichment factors of 2.82 (AAV8) and 5.61 (AAV9). These results demonstrate that the number and position of His residues in mixed-mode peptide ligands provide a pH-tunable binding mechanism capable of discriminating capsid quality attributes within a single chromatographic step, a capability not offered by conventional immunoaffinity ligands.

The structural framework developed in this study has critical implications for the design of next-generation ligands for the chromatographic purification and enrichment of gene therapies. Serotype-agnostic ligands should target conserved pH-responsive hotspots rich in polar, aromatic, and hydrogen bond-forming residues that are located within the chain A binding surface and adjacent interchain interfaces, and are conserved across serotypes. Serotype-selective ligands, conversely, should be engineered to exploit differences in the amplitude and spatial coupling of remodeling, such as the distributed interchain response of AAV8 versus the focused, chain A-centered response of AAV9. We notice that our approach of conducting MD simulations on pentameric assemblies rather than full capsids enables a tractable pH-resolved sampling, but may not fully capture long-range allosteric effects operating across the virion. Future work should extend these analyses to additional clinically relevant serotypes, incorporate the simulation of transgene-containing vs. empty capsids at the structural level, and use the identified pH-responsive hotspots as input for de novo computational peptide design. These efforts will bridge the gap between structural biology and the rational development of programmable, quality-enriching capture reagents for gene therapy manufacturing.

These findings translate into four operational rules for the design of peptide ligands for AAV capture, derived from three mechanistically contrasting ligands selected from a larger ensemble in a separate design study [17]. First, a cluster of histidines confers selectivity for transduction-competent capsids near neutral pH, because partial protonation one unit above the imidazole pKa generates an electrostatic and hydrogen-bonding array finely enough graded to discriminate between closely related capsid surface states; TYHHHHII exemplifies this rule, and loses the discrimination entirely at pH 9 once the cluster is fully deprotonated. Second, at least one permanently cationic anchor—an arginine, or a residue of comparable pKa—confers robustness across a broad pH window by providing an electrostatic contact that does not depend on protonation state; TTFRAHHI exemplifies this rule and delivers the highest recovery of the three ligands at both pH 7 and pH 9. Third, aromatic and hydrophobic residues such as Tyr, Phe, Ile, and Val should be used to reinforce binding through non-electrostatic contacts once recognition has occurred, rather than to drive recognition itself; RVVAVYRI, which relies predominantly on this mode, binds stably but without selectivity. Fourth, the spacing and local context of these residues should be matched to the serotype-specific remodeling of the chain A and interchain epitope—the broadly distributed response of AAV8 against the spatially focused response of AAV9—rather than treated as a universal AAV-binding motif.

In summary, this study isolates the effect of ligand sequence and solution pH, while ligand display and chromatographic substrate were held constant; their optimization, together with resin lifetime and benchmarking against commercial resins, is addressed in a companion study [17]. The chromatographic data report apparent, avidity-dominated capture, and the design rules proposed above, derived from three mechanistically distinct ligands, invite validation against a broader library, as does causal testing of the residue-level mechanism by targeted mutagenesis with appropriate assembly controls.

## 5. Patent

A.M., B.B., and S.M. are named inventors on a U.S. provisional patent application (No. 63/101,077, filed 29 June 2026) titled “Compositions and Methods for Enhanced Purification and Enrichment of Gene-Loaded Adeno-Associated Viral Vectors”, filed by NC State University and related to the technology described in this manuscript.

## Data Availability

The original contributions presented in this study are included in the article/Supplementary Material. Further inquiries can be directed to the corresponding author(s).

## Supplementary Materials

The following supporting information can be downloaded at: https://www.mdpi.com/article/10.3390/pharmaceutics18091053/s1, Buffer composition and pK_a_ values and ionic strength analysis of buffer selection (Tables S1 and S2); yield, NCP reduction, and transduction activity enrichment factors for AAV8 (Table S3) and AAV9 (Table S4); pH-dependent net charge profiles of the three peptide ligands (Figure S1); SEC-HPLC analysis of chromatographic fractions containing AAV8 (Figures S2 and S3) and AAV9 (Figures S4 and S5); inter-residue contact dynamics and pore coupling in AAV8 and AAV9 (Figure S6); solvent-accessible surface area comparison across pH conditions (Figure S7); electrostatic potential comparison across pH conditions (Figure S8); coupling between solvent accessibility, electrostatics, and contact reorganization at peptide-binding surfaces (Figure S9). Figure S10. Convergence of the molecular dynamics trajectories.
