# Supplementary material for: Probing the Capsid: pH-Driven Gating at the AAV 5-Fold Pore and Its Role in Peptide Ligand Binding
Source: Pharmaceutics. 2026 Aug 25;18(9):1053. doi: 10.3390/pharmaceutics18091053 (PMC13610328; doi:10.3390/pharmaceutics18091053)
Supplement: Supplementary file 1 [file pharmaceutics-18-01053-s001.zip › pharmaceutics-4468856-supplementary.pdf]

Table S1. *Buffers utilized in this study.*

pH	Buffer	pKa	structure
5	Sodium Acetate	NA	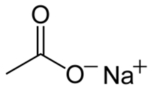
7	Bis-Tris	pH range = 5.8 – 7.2<br>pKa = 6.46 @ 25°C	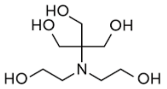
9	Bis-Tris Propane (BTP)	pH range = 6.0 – 9.5<br>pKa_1 = 6.8 @ 25°C<br>pKa_2 = 9.0 @ 25°C	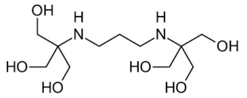

Table S2. *Ionic strength analysis on buffer selection.*

pH	Condition	Buffer IS (mM)	NaCl IS (mM)	Total IS (mM)
5	10mM NaOAc + 20mM NaCl	10.0	20.0	30.0
7	10mM Bis-Tris + 20mM NaCl	2.2	20.0	22.2
9	10mM Bis-Tris Propane + 20mM NaCl	5.1	20.0	25.1

Table S3. *Yield, HCP reduction, and transduction activity enrichment factor measured in eluates produced by purifying AAV8 from a clarified HEK293 cell lysate using RVVAVYRI-, TTFRAHHI-, and TYHHHHII-functionalized resins. The capsid and HCP titers were measured by AAV8-specific and HEK293 HCP-specific ELISA kits, while the titer of cell-transducing units was measured by fluorescence flow cytometry. Yield and HCP reduction are expressed as percentage and log reduction value (LRV), respectively; the enrichment factor is defined as the ratio of specific transduction activity (TU/capsid) in the eluate to that in the load. Each chromatographic condition was performed as a single run; the capsid ELISA, HCP ELISA, SEC-HPLC, and transduction assay quantifications were performed in technical replicate (n = 3), and the values reported are the means of those replicates.*

AAV8	SEC-HPLC			Capsid ELISA	HCP ELISA	Transduction assay
pH_sequence	Yield (Elution)	BOUND (Elution + Strip)	UNBOUND (LFT + WASH)	Yield (Elution)	HCP LRV	Activity Enrichment Factor
pH5_RVVAVYRI	0.27 ± 0.03%	9.92 ± 2.46%	83.88 ± 3.02%	3.75 ± 1.34%	N/A	N/A
pH5_TTFRAHHI	0.39 ± 0.05%	2.67 ± 1.72%	88.31 ± 2.56%	2.98 ± 1.75%	N/A	N/A
pH5_TYHHHHII	0.26 ± 0.12%	1.52 ± 1.07%	88.24 ± 5.89%	0.78 ± 0.17%	N/A	N/A
pH7_RVVAVYRI	18.96 ± 1.35%	52.95 ± 1.18%	0.70 ± 0.14%	24.29 ± 3.67%	1.24 ± 0.08	1.61 ± 0.31
pH7_TTFRAHHI	49.64 ± 1.31%	63.74 ± 2.06%	3.33 ± 0.28%	44.59 ± 3.12%	1.49 ± 0.04	1.16 ± 0.18
pH7_TYHHHHII	29.55 ± 1.17%	30.25 ± 2.98%	44.26 ± 1.69%	35.08 ± 1.53%	1.60 ± 0.09	2.82 ± 0.26
pH9_RVVAVYRI	32.01 ± 1.29%	76.90 ± 2.01%	5.88 ± 1.07%	26.90 ± 2.17%	1.18 ± 0.05	1.29 ± 0.27
pH9_TTFRAHHI	60.94 ± 2.08%	64.90 ± 3.19%	1.91 ± 0.75%	50.16 ± 1.87%	1.37 ± 0.11	1.10 ± 0.17
pH9_TYHHHHII	0.36 ± 0.19%	0.71 ± 0.37%	75.67 ± 4.58%	0.09 ± 0.05%	N/A	N/A

Table S4. *Yield, HCP reduction, and transduction activity enrichment factor measured in eluates produced by purifying AAV9 from a clarified HEK293 cell lysate using RVVAVYRI-, TTFRAHHI-, and TYHHHHII-functionalized resins. The capsid and HCP titers were measured by AAV9-specific and HEK293 HCP-specific ELISA kits, while the titer of cell-transducing units was measured by fluorescence flow cytometry. Yield and HCP reduction are expressed as percentage and log reduction value (LRV), respectively; the enrichment factor is defined as the ratio of specific transduction activity (TU/capsid) in the eluate to that in the load. Each chromatographic condition was performed as a single run; the capsid ELISA, HCP ELISA, SEC-HPLC, and transduction assay quantifications were performed in technical replicate (n = 3), and the values reported are the means of those replicates.*

AAV9	SEC-HPLC			Capsid ELISA	HCP ELISA	Transduction assay
pH_sequence	Yield (Elution)	BOUND (Elution + Strip)	UNBOUND (LFT + WASH)	Yield (Elution)	HCP LRV	Activity Enrichment Factor
pH5_RVVAVYRI	7.94 ± 0.83%	17.18 ± 1.30%	32.73 ± 1.89%	5.71 ± 1.67%	0.97 ± 0.12	0.43 ± 0.08
pH5_TTFRAHHI	8.27 ± 0.56%	10.31 ± 0.97%	40.72 ± 2.15%	11.80 ± 1.42%	0.82 ± 0.05	0.20 ± 0.14
pH5_TYHHHHII	4.36 ± 0.73%	7.10 ± 0.78%	36.80 ± 2.03%	3.27 ± 0.65%	0.68 ± 0.04	0.51 ± 0.12
pH7_RVVAVYRI	10.22 ± 0.68%	34.94 ± 1.59%	48.79 ± 2.39%	6.60 ± 0.67%	0.58 ± 0.05	3.39 ± 0.24
pH7_TTFRAHHI	21.78 ± 0.92%	31.11 ± 1.84%	61.73 ± 2.74%	30.43 ± 1.52%	1.03 ± 0.09	3.21 ± 0.18
pH7_TYHHHHII	14.39 ± 0.83%	20.51 ± 1.45%	60.82 ± 2.71%	21.90± 2.07%	0.72 ± 0.05	5.61 ± 0.27
pH9_RVVAVYRI	48.96 ± 1.53%	77.54 ± 2.12%	24.66 ± 1.61%	28.27 ± 2.41%	1.51 ± 0.03	2.66 ± 0.39
pH9_TTFRAHHI	65.52 ± 1.45%	81.00 ± 2.57%	16.80 ± 1.29%	62.20 ± 3.87%	1.43 ± 0.07	2.04 ± 0.17
pH9_TYHHHHII	0.47 ± 0.09%	0.47 ± 0.09%	89.94 ± 1.40%	0.05 ± 0.04%	N/A	N/A

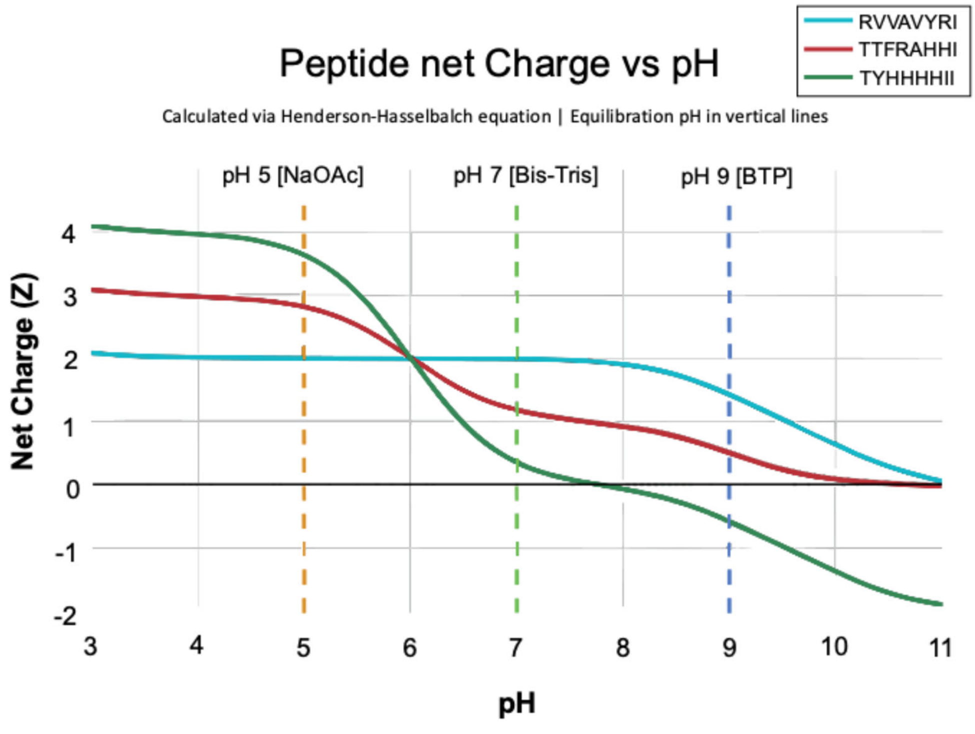

Figure S1. *pH-dependent net charge profiles of three candidate peptide ligands calculated by the Henderson-Hasselbalch equation. Net charge (Z) as a function of pH (3 - 11) is shown for RVVAVYRI (cyan), TTFRAHHI (red), and TYHHHHII (green), with vertical dashed lines marking the equilibration pH values used in chromatography experiments: pH 5 (sodium acetate, NaOAc; orange), pH 7 (Bis-Tris; green), and pH 9 (BTP; blue).*

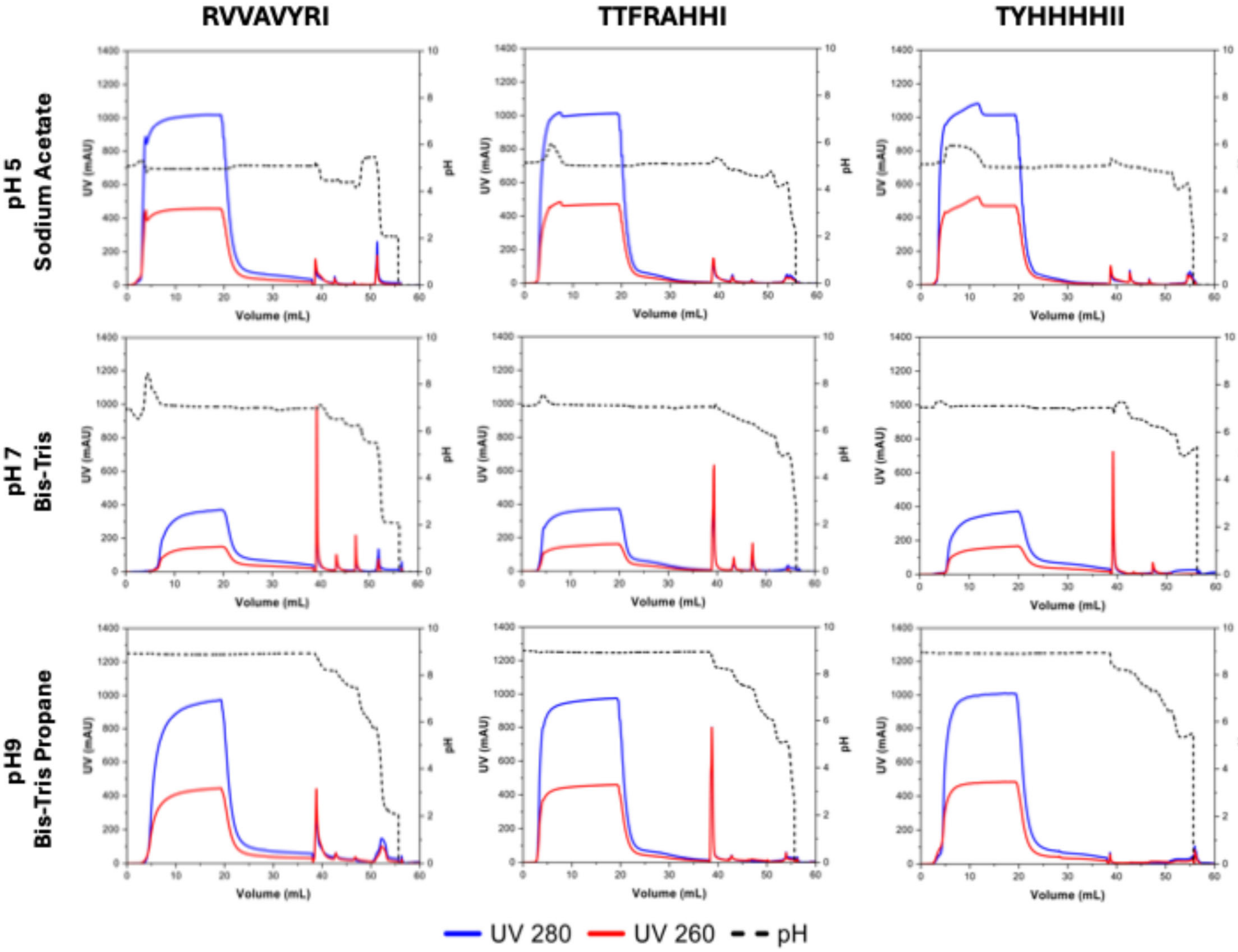

Figure S2. *Chromatograms obtained by purifying AAV8 from a clarified HEK293 cell lysate (AAV load ~6.0·10*^{13} *vps, HCP titer: 0.28 mg/mL) using RVVAVYRI-, TTFRAHHI-, and TYHHHHII-functionalized resins following the procedure described in the Materials and Methods section 4.3.*

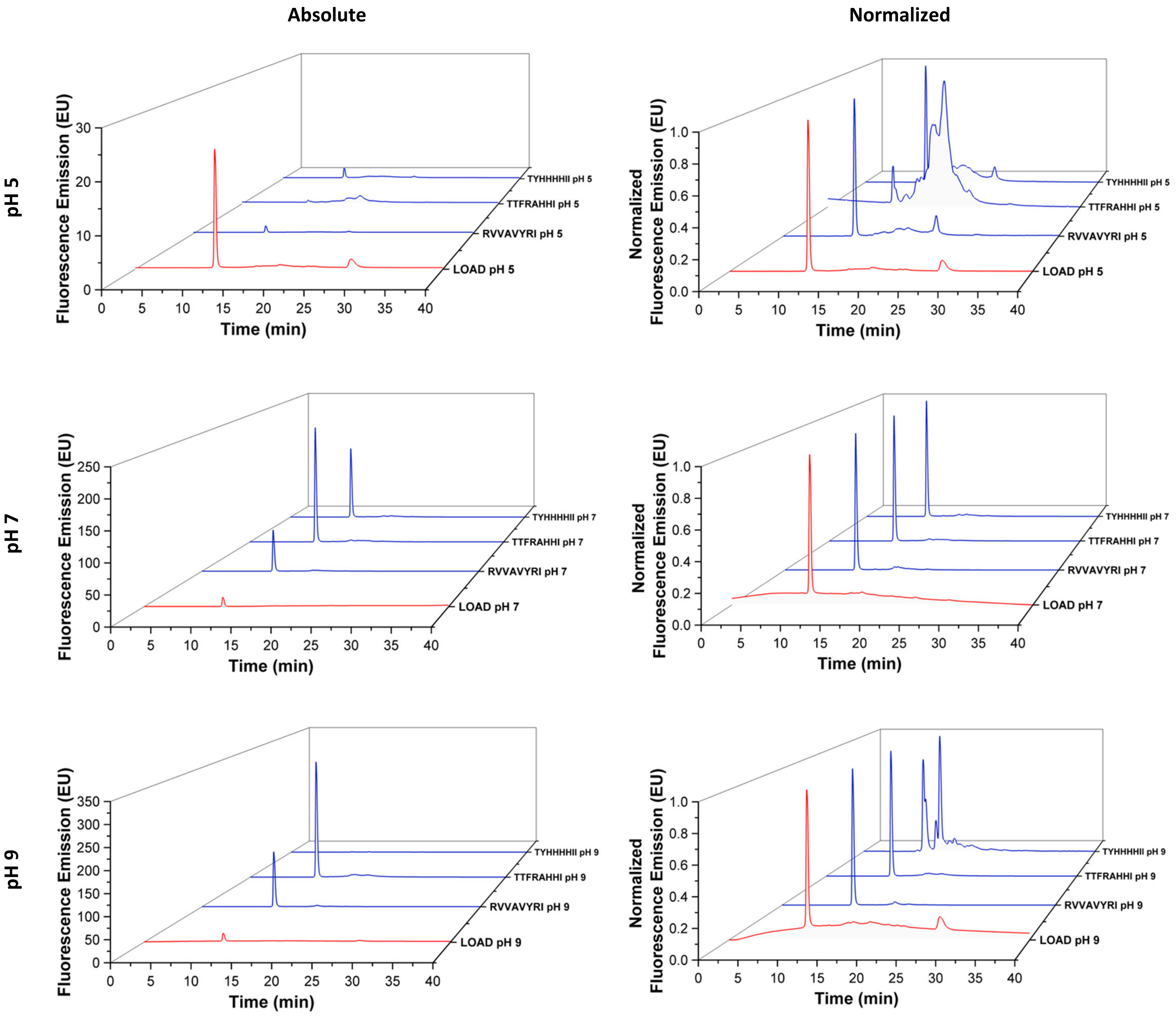

Figure S3. *Absolute and normalized SEC-HPLC analysis of clarified HEK293 cell lysates (red) at pH 5, 7, 9, and the corresponding eluates (blue) obtained by purifying AAV8 from a clarified HEK293 cell lysate using RVVAVYRI-, TTFRAHHI-, and TYHHHHII-functionalized resins. The chromatograms were produced by monitoring the effluents via fluorescence spectrophotometry (exc/em = 280/350 nm).*

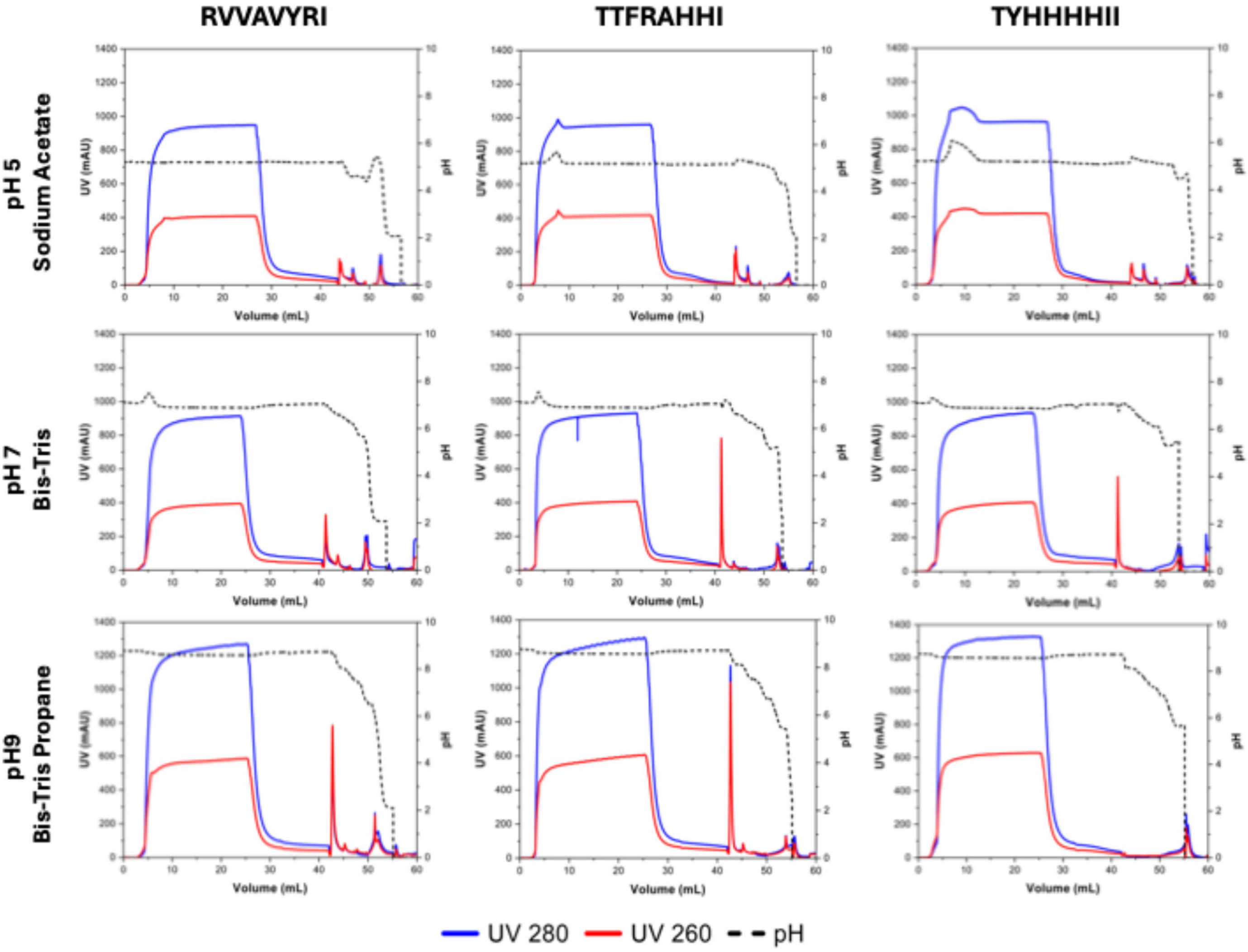

Figure S4. *Chromatograms obtained by purifying AAV9 from a clarified HEK293 cell lysate (AAV load ~6.9·10*^{13} *vps, HCP titer: 0.32 mg/mL) using RVVAVYRI-, TTFRAHHI-, and TYHHHHII-functionalized resins following the procedure described in the Materials and Methods section 4.*

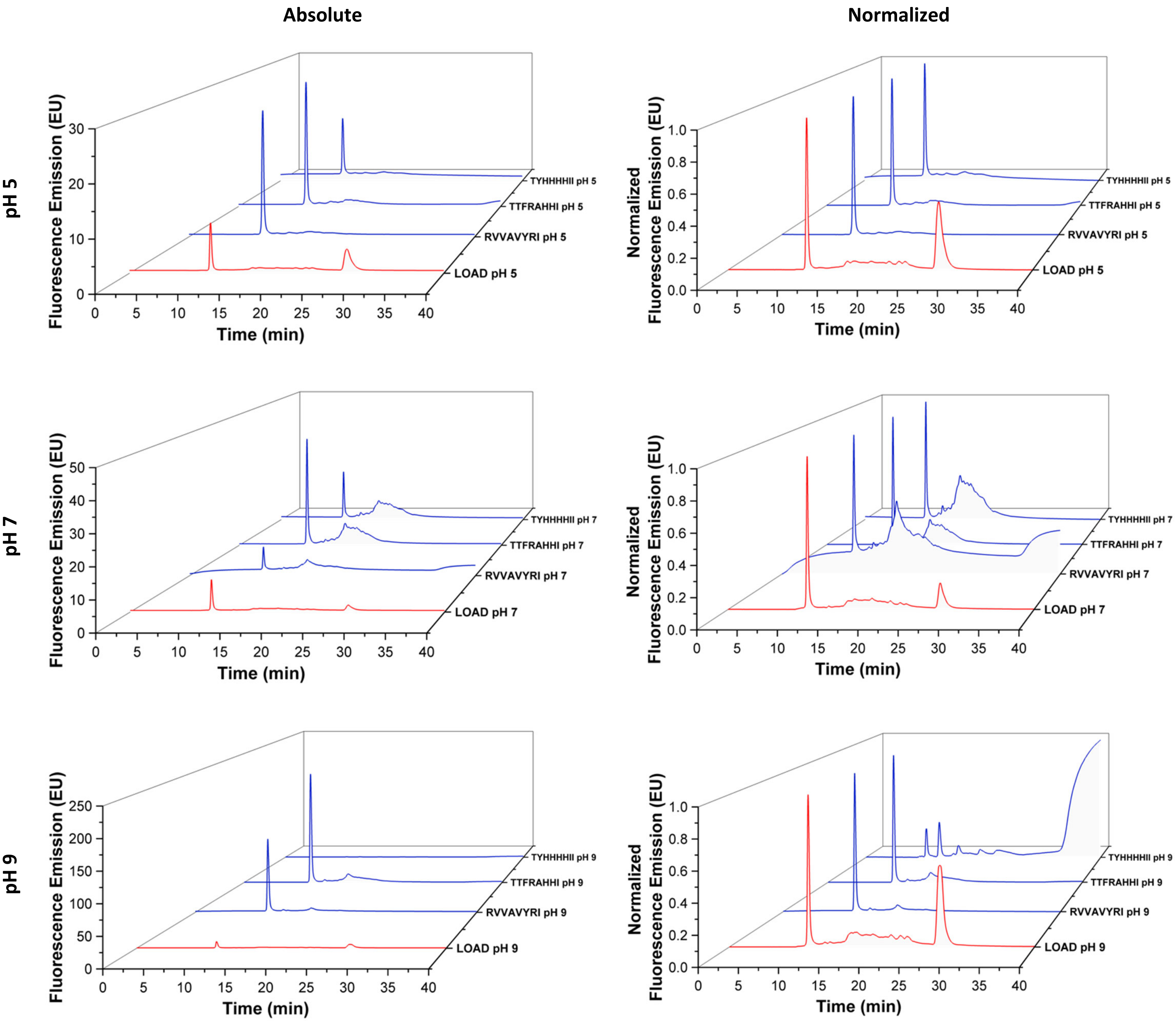

Figure S5. *Absolute and normalized SEC-HPLC analysis of clarified HEK293 cell lysates (red) at pH 5, 7, 9, and the corresponding eluates (blue) obtained by purifying AAV9 from a clarified HEK293 cell lysate using RVVAVYRI-, TTFRAHHI-, and TYHHHHII-functionalized resins. The chromatograms were produced by monitoring the effluents via fluorescence spectrophotometry (exc/em = 280/350 nm).*

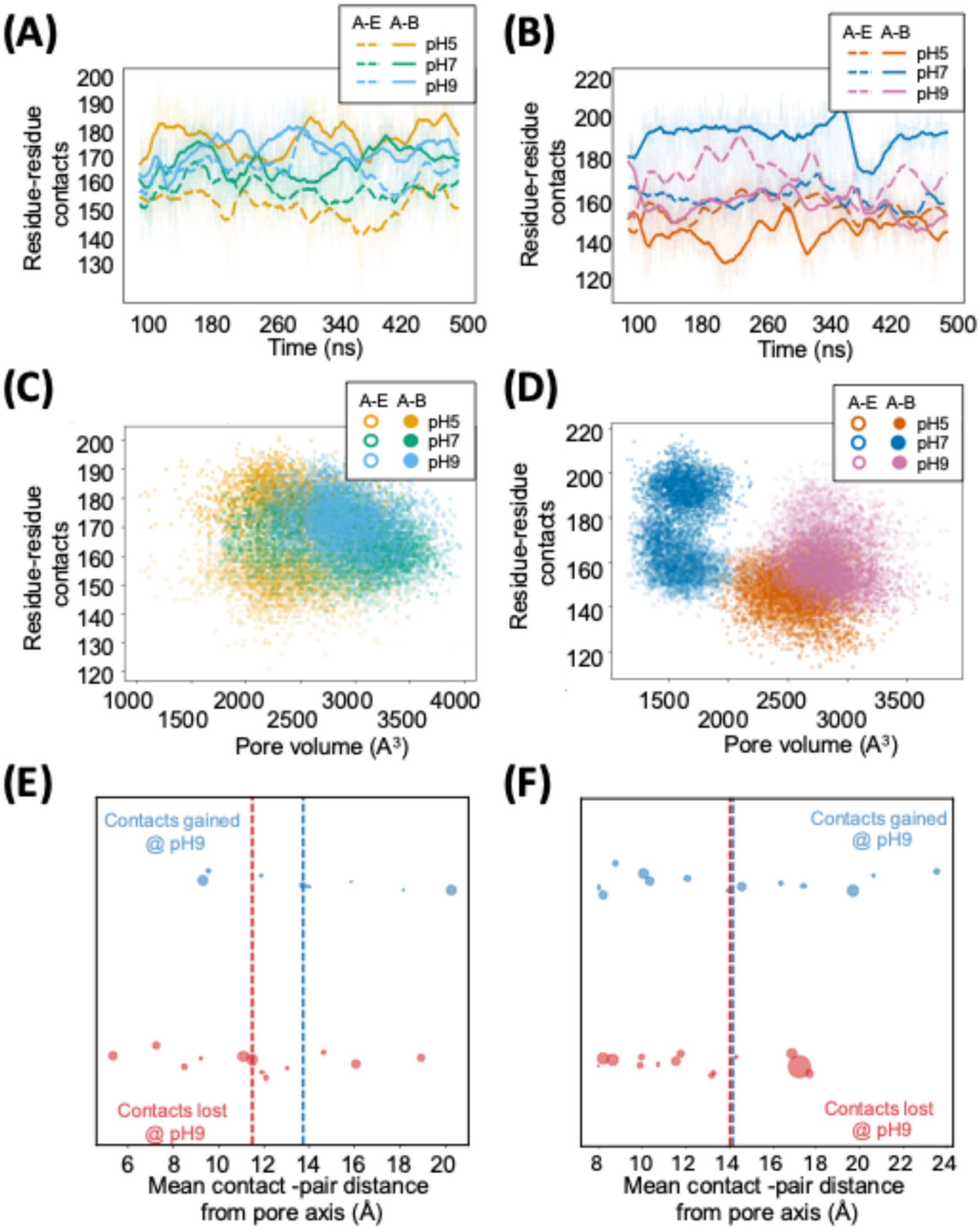

Figure S6. Contact dynamics and pore coupling in AAV8 and AAV9. *Panels A–B, time–resolved residue–residue contact counts combining A–E (dashed lines) and A–B (solid lines) interfaces for AAV8 (A) and AAV9 (B) across pH conditions (pH 5, 7, and 9). Thicker lines represent running averages, and shaded regions indicate fluctuations over time. Panels C–D, relationship between residue–residue contacts and pore volume for AAV8 (C) and AAV9 (D). Each point represents a frame from the MD trajectory, colored by pH condition. Panels E–F, spatial distribution of contact changes relative to the pore axis for AAV8 (E) and AAV9 (F). Each point represents a contact pair, plotted by its mean distance from the pore axis, with marker size proportional to the magnitude of the contact change (Δ occupancy).*

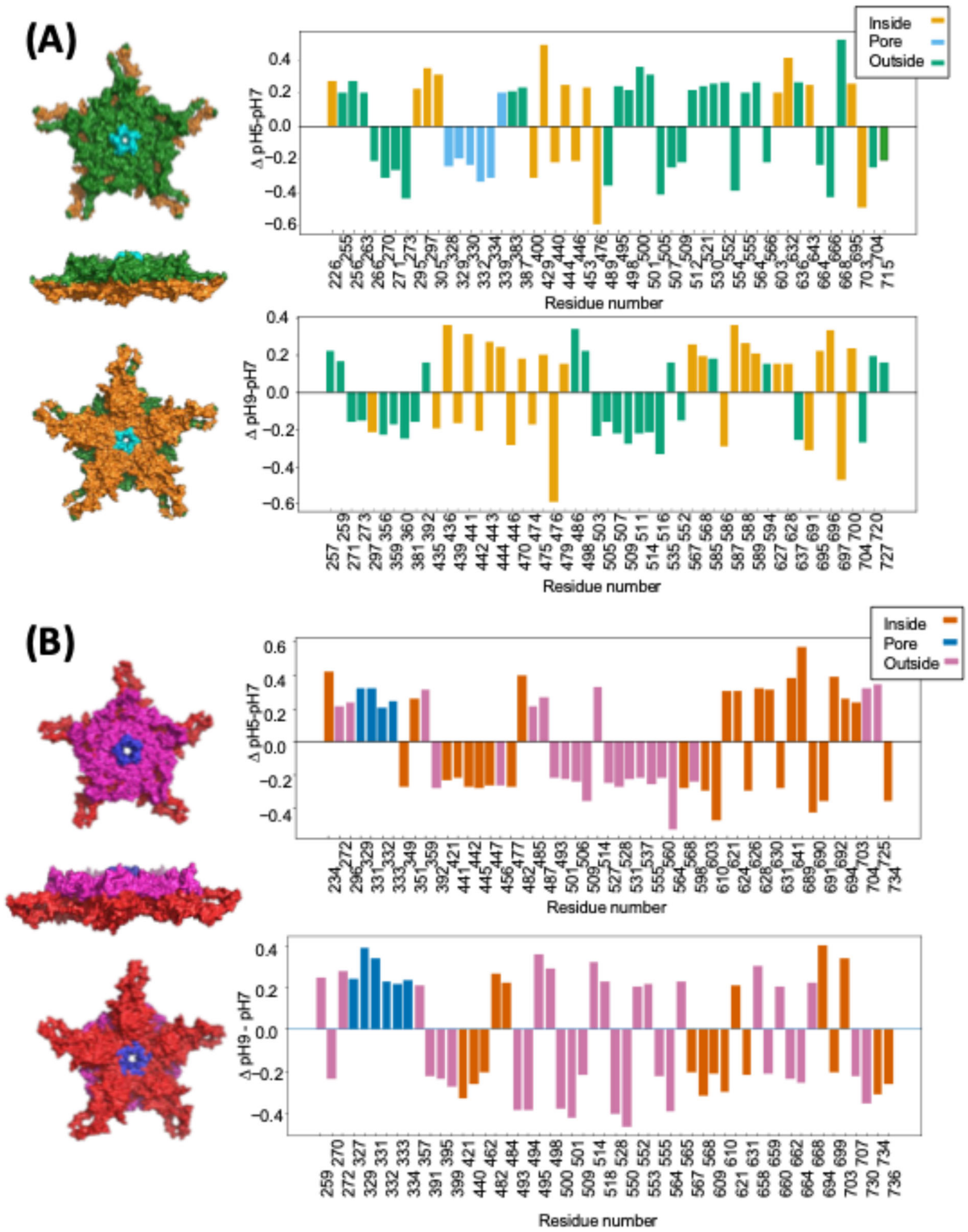

Figure S7. Comparison of SASA vs pH. *Definition of residue classifications for outside (outer surface – green and magenta), pore (cyan and blue), and inside (inner surface – orange and red) regions of the capsid. Panel A, represents the AVV8 definition of pore, outside, and inside residues. The same color coding is used on the bar graph of the top 10% of residues that changed with pH: top – pH 5-pH 7; bottom – pH 9-pH 7. Panel B represents the AVV9 definition of pore, outside, and inside residues. The same color coding is used in the bar graph of the top 10% residues that changed with pH: top – pH 5-pH 7; bottom – pH 9-pH 7.*

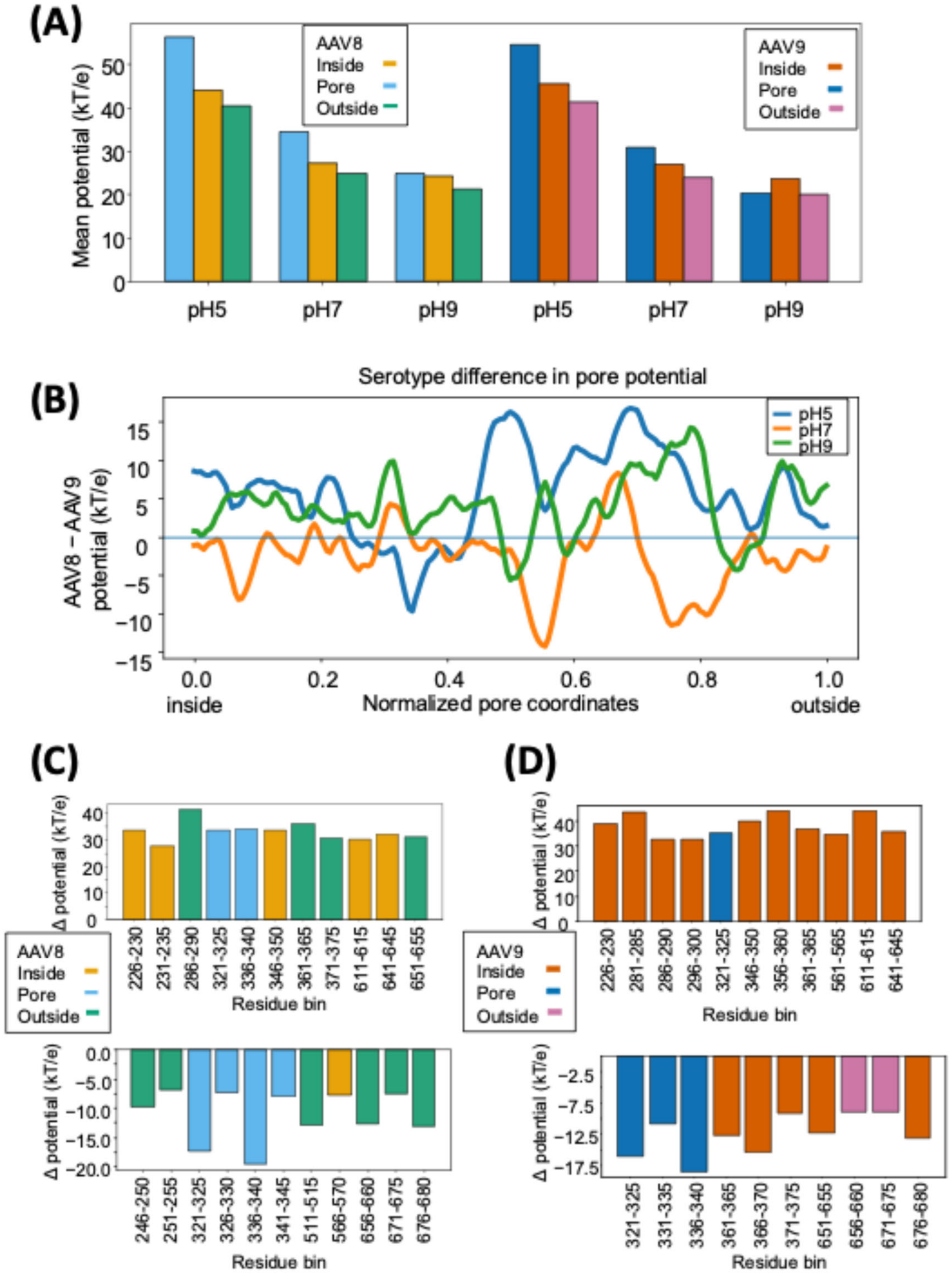

Figure S8. AAV8 and AAV9 electrostatics comparison. *Panel A, mean electrostatic potential (kT/e) for interior capsid (orange and red), pore (cyan and blue), and exterior (green and pink) surface regions in AAV8 (left) and AAV9 (right) at pH 5, 7, and 9. Panel B, serotype-specific differences in electrostatic potential along the normalized pore axis (AAV8 − AAV9). The normalized pore coordinate represents position along the fivefold pore axis, scaled from 0 at the capsid interior (lumen-facing side; residues 220–224) to 1 at the exterior-facing end of the pore (top/pore-lining region; residues ~333–339). Panels C–D, top 10% of residue bins ranked by electrostatic change (Δ potential relative to pH 7) for AAV8 (C) and AAV9 (D), separated into pH 5-7 (top) and pH 9-7 (bottom) deviations. Bars are colored by structural region (inside capsid, pore, outside surface – same as above). Labels are provided for clarity.*

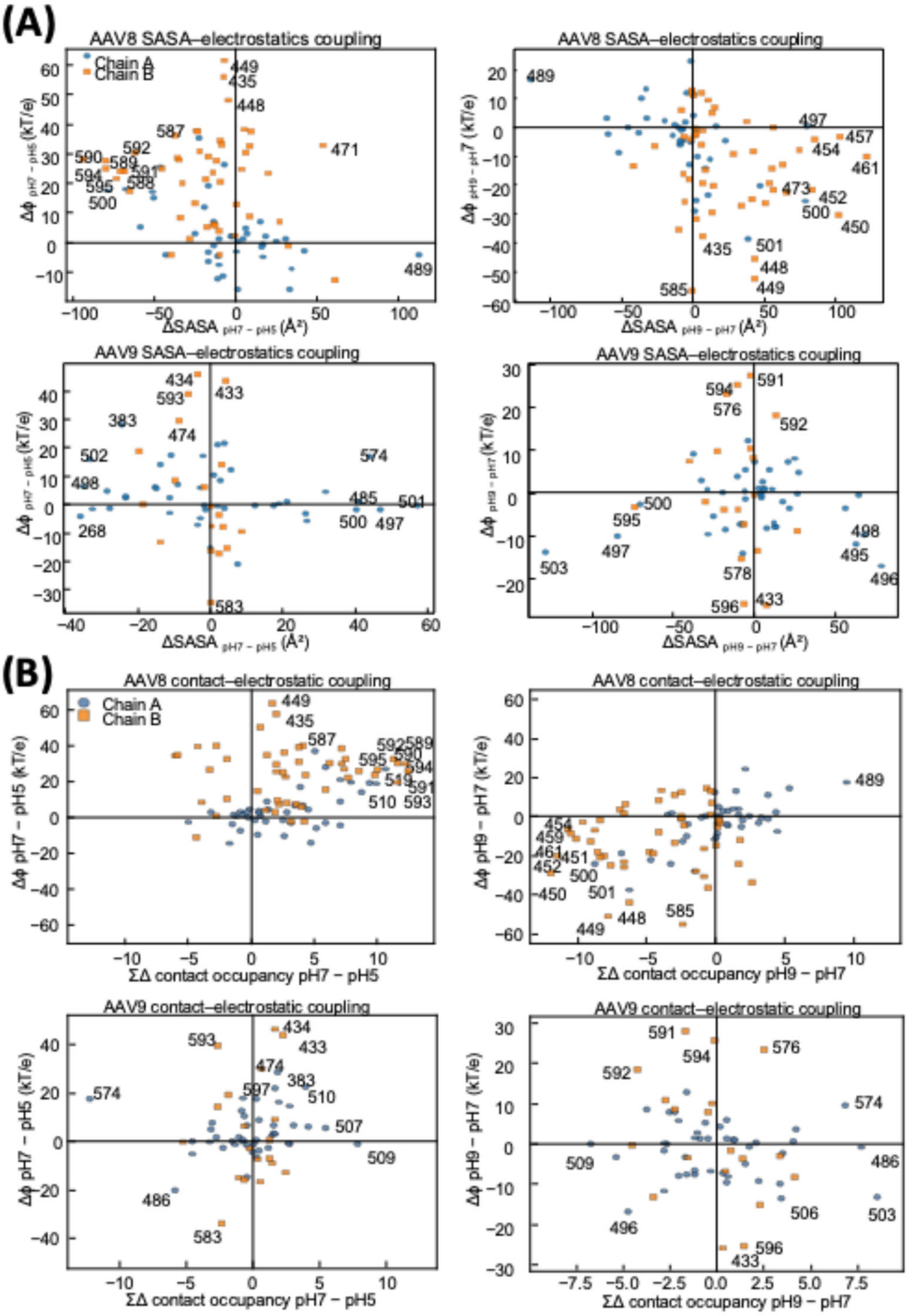

Figure S9. Coupling between solvent accessibility, electrostatic remodeling, and contact reorganization within the peptide-binding surfaces of AAV8 and AAV9. *(A) Relationship between residue-level changes in solvent accessible surface area (ΔSASA) and electrostatic potential (Δϕ) across pH transitions for AAV8 (top) and AAV9 (bottom). Left panels show the pH7−pH5 transition, and right panels show the pH9−pH7 transition. Each point represents a binding-site residue from chain A (blue circles) or neighboring interfacial chains (orange squares). Residues exhibiting the largest coupled changes are labeled. (B) Relationship between residue-level contact remodeling (ΣΔ contact occupancy) and electrostatic potential changes (Δϕ) across pH transitions for AAV8 (top) and AAV9 (bottom). Left panels show the pH7−pH5 transition, and right panels show the pH9−pH7 transition. Contact remodeling values represent the sum of occupancy changes for all residue–residue contacts involving each potential binding-site residue.*

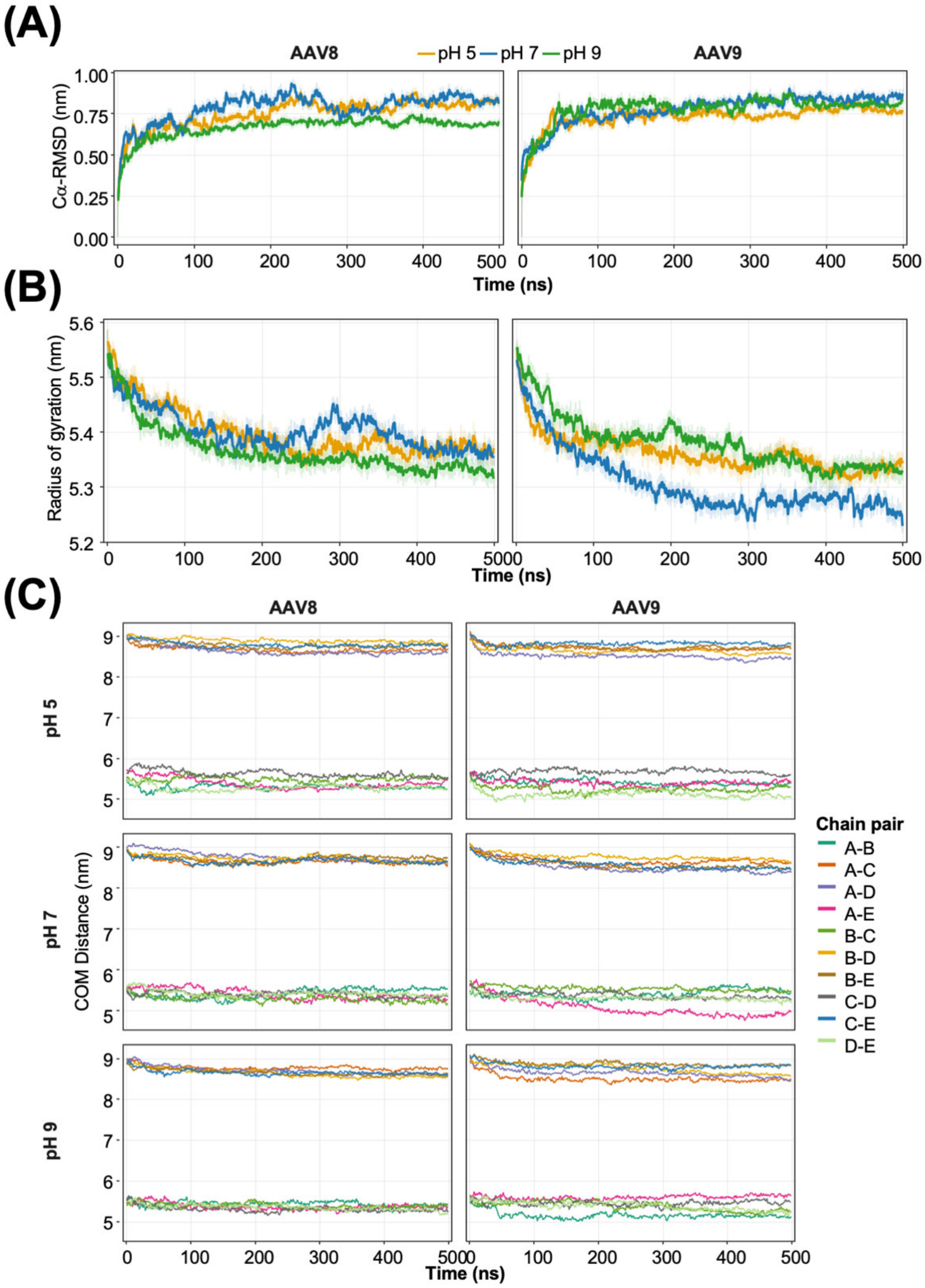

Figure S10. *Convergence of the molecular dynamics trajectories. (A) Cα root mean square deviation (RMSD) relative to the energy-minimized starting structure as a function of simulation time for the AAV8 (left) and AAV9 (right) 5-mer assemblies at pH 5, pH 7, and pH 9. (B) radius of gyration (R_g) of the pentameric assembly over the same trajectories. (C) displacement of the assembly center of mass (COM) over the same trajectories. All three metrics plateau within the first 100 ns in every system; the first 100 ns of each trajectory were therefore discarded as equilibration, and all analyses reported in the manuscript were performed on the final 400 ns. Because molecular dynamics trajectories sample a configurational ensemble rather than independent biological realizations, these convergence metrics – together with the agreement of the independent structural descriptors reported in the manuscript – constitute the basis on which the robustness of the computational results is assessed.*
